# Broad coverage of neutralization-resistant SIV strains by second-generation SIV-specific antibodies targeting the region involved in binding CD4

**DOI:** 10.1371/journal.ppat.1010574

**Published:** 2022-06-16

**Authors:** Hugh C. Welles, Hannah A. D. King, Leonard Nettey, Nicole Cavett, Jason Gorman, Tongqing Zhou, Yaroslav Tsybovsky, Renguang Du, Kaimei Song, Richard Nguyen, David Ambrozak, Amy Ransier, Chaim A. Schramm, Nicole A. Doria-Rose, Adrienne E. Swanstrom, James A. Hoxie, Celia LaBranche, David C. Montefiori, Daniel C. Douek, Peter D. Kwong, John R. Mascola, Mario Roederer, Rosemarie D. Mason

**Affiliations:** 1 Vaccine Research Center, National Institutes of Health, Bethesda, Maryland, United States of America; 2 U.S. Military HIV Research Program, Walter Reed Army Institute of Research, Silver Spring, Maryland, United States of America; 3 Henry M. Jackson Foundation for the Advancement of Military Medicine, Bethesda, Maryland, United States of America; 4 Vaccine Research Center Electron Microscopy Unit, Cancer Research Technology Program, Leidos Biomedical Research, Inc., Frederick National Laboratory for Cancer Research, Frederick, Maryland, United States of America; 5 AIDS and Cancer Virus Program, Frederick National Laboratory for Cancer Research, Frederick, Maryland, United States of America; 6 Department of Medicine, Perelman School of Medicine, University of Pennsylvania, Philadelphia, Pennsylvania, United States of America; 7 Department of Surgery, Duke University Medical Center, Durham, North Carolina, United States of America; Emory University, UNITED STATES

## Abstract

Both SIV and SHIV are powerful tools for evaluating antibody-mediated prevention and treatment of HIV-1. However, owing to a lack of rhesus-derived SIV broadly neutralizing antibodies (bnAbs), testing of bnAbs for HIV-1 prevention or treatment has thus far been performed exclusively in the SHIV NHP model using bnAbs from HIV-1-infected individuals. Here we describe the isolation and characterization of multiple rhesus-derived SIV bnAbs capable of neutralizing most isolates of SIV. Eight antibodies belonging to two clonal families, ITS102 and ITS103, which target unique epitopes in the CD4 binding site (CD4bs) region, were found to be broadly neutralizing and together neutralized all SIV strains tested. A rare feature of these bnAbs and two additional antibody families, ITS92 and ITS101, which mediate strain-specific neutralizing activity against SIV from sooty mangabeys (SIVsm), was their ability to achieve near complete (i.e. 100%) neutralization of moderately and highly neutralization-resistant SIV. Overall, these newly identified SIV bnAbs highlight the potential for evaluating HIV-1 prophylactic and therapeutic interventions using fully simian, rhesus-derived bnAbs in the SIV NHP model, thereby circumventing issues related to rapid antibody clearance of human-derived antibodies, Fc mismatch and limited genetic diversity of SHIV compared to SIV.

## Author summary

Antibodies that can bind to a virus and block infection are called neutralizing antibodies. In human immunodeficiency virus (HIV), antibodies that can block multiple strains of HIV circulating globally are known as broadly neutralizing antibodies. These broadly neutralizing antibodies have the potential to be used to treat HIV-infected individuals or to prevent HIV infection altogether. However, before these broadly neutralizing antibodies can safely be used in humans, they must first be tested in monkeys. Simian immunodeficiency virus (SIV), which causes an acquired immunodeficiency syndrome (AIDS)-like illness in Asian macaques that is similar to AIDS in humans, makes it possible to perform pre-clinical testing in monkeys but requires the use of antibodies that are specific to SIV. Another important consideration for testing antibodies in monkeys is that, like HIV, there are many different strains of SIV and some of these are highly resistant to neutralization. In this study we used blood from SIV-infected macaques to isolate several antibodies that can neutralize multiple strains of SIV, including strains that are typically resistant to neutralization. These SIV broadly neutralizing antibodies closely resemble HIV broadly neutralizing antibodies, making it possible now to test antibody-based vaccines or therapies for HIV using the SIV nonhuman primate model of AIDS.

## Introduction

To date, numerous HIV-1-specific broadly neutralizing antibodies (bnAbs) have been isolated and extensively characterized [1–3]; however, antibody-mediated therapies to treat or prevent HIV-1 infection have yet to be developed [4]. Key to developing such antibody-based therapies will be careful consideration of distinct HIV-1 bnAb virus neutralization breadth and potency profiles, virus escape patterns, *in vivo* pharmacokinetics, tissue distribution and safety profiles [5–11]. Thus, comprehensive testing of individual or combination bnAbs to treat or prevent HIV-1 infection will likely be needed before adopting HIV-1 bnAbs for clinical use. Pre-clinical testing in non-human primates (NHP) will best inform the selection of bnAbs most likely to achieve optimal efficacy for the treatment and prevention of HIV-1 infection.

We previously reported on the isolation of a broad array of SIV-specific monoclonal antibodies (mAbs) targeting multiple regions of SIV Env [12]. These include SIV mAbs targeting the CD4-binding site (CD4bs), CD4-induced (CD4i) site, variable loops 1, 2 and 3 (V1, V2 and V3) and the high-mannose patch (HMP) of SIV Env. Several of these mAbs cross-neutralized multiple strains of SIV and HIV-2 isolate 7312A. However, a peculiar and consistent characteristic of these SIV neutralizing antibodies (nAbs), as with sera from vaccinated or SIV-infected macaques, is an inability to completely neutralize moderately or highly neutralization-resistant isolates of SIV [12–15]. Specifically, even the broadest of SIV-neutralizing mAbs fail to neutralize highly neutralization-resistant SIV strains and only partially or incompletely neutralize moderately neutralization-resistant SIV strains, even at the highest nAb concentrations tested. This neutralization plateau effect has been reported for both SIV and HIV-1 [16]. Whereas the neutralization plateau observed with some HIV-1 bnAbs against certain isolates has been attributed, in part, to glycan heterogeneity of particular Env epitopes, the neutralization plateau observed for SIV neutralizing antibodies (nAbs) against specific strains of SIV is marked and consistent and cannot be solely ascribed to glycans. Despite this neutralization resistance, the SIV NHP model complements the SHIV model for determining the efficacy of mAb-based HIV-1 interventions since the genetic diversity of naturally circulating SIVmac subtypes is comparable to the genetic diversity of HIV-1 circulating strains [12,14,17,18]. A paucity of well-characterized rhesus SIV bnAbs suitable for preclinical studies of HIV-1 bnAbs is a major obstacle to using the SIV NHP challenge model to evaluate HIV-1 mAb-based interventions. Here, we sought to isolate rhesus-derived SIV bnAbs that could mediate neutralization breadth similar to HIV-1 bnAbs, and, more importantly, overcome the neutralization plateau effect that is a hallmark of virtually all SIV nAbs isolated thus far.

## Materials and methods

### Ethics statement

Animals were handled in accordance with the standards of the American Association for the Accreditation of Laboratory Animal Care (AAALAC) and meet NIH standards as set forth in the Guidelines for Care and Use of Laboratory Animals. Animal experiments were approved by the Institutional Animal Care and Use Committee of Tulane University (protocol P0245 and P0245R). The Tulane National Primate Research Center (TNPRC) is fully accredited by AAALAC International, Animal Welfare Assurance No. A4499-01.

### Indian-origin rhesus macaque specimens

SIV-positive plasma and peripheral blood mononuclear cells (PBMC) from animal 8E-9 were obtained from a previously completed animal study protocol evaluating the effect of TRIM5 genotype on mucosal acquisition of SIVsmE660 [19]. Plasma and PBMC from animal IK16, a rhesus macaque vaccinated with iMac-ΔD385, a non-CD4 tropic variant of SIVmac239 [20], and challenged with an E660 swarm virus (provided by Vanessa Hirsch, NIAID, NIH), were provided by James Hoxie (Univ. of Pennsylvania) (S1 Fig).

### Virus neutralization and competition assays

Plasmid DNA encoding SIV gp160 was used in combination with a luciferase reporter plasmid containing the essential HIV structural genes to produce SIV Env-pseudo-typed viruses as described previously [12]. Plasmids encoding SIV gp160 for clones SIVsmE660.CP3C.A8 (GenBank accession number FJ578816), SIVsmE660.CR54.2A5 (GenBank accession number FJ578939), SIVmac251.H9.15, SIVmac251.30, SIVmac239, SIVsmE660.11 [21], SIVmac239.cs.23 [22], SIVmac251.6 [22] and SIVmac251.cs.41 [23] were provided by David Montefiori. Plasmids for SIVmac251 clones (RZu4_16Apr09_EnvPL1.1 and RZj5 9Apr09 ENVPL2.1), and SIVsm clones (FFv 18Nov04 ENVPL2.1, FJv 15Nov06 ENVPL2.1, FWk 12Aug04 ENVPL4.1 and RSo8 17Jan06 ENVPL1.1) were kindly provided by Cynthia Derdeyn [24]. Full-length infectious molecular clones of transmitted/founder viruses SIVsmm lineage 1 (RM174.V1,V2,V3.tf), 5 (FTq) and “outlier” (SL92b) were derived by plasma inoculation of naïve rhesus macaques (H.L., G.M.S.; GenBank accession numbers KU182919-KU182923) and represent examples of highly diverse naturally-occurring strains of SIVsmm [25]. Virus neutralization was measured using single round infection of TZM-bl target cells by SIV Env pseudo-typed virus or replication-competent viruses i.e., infectious molecular clones (IMC) in the presence of the protease inhibitor indinavir, as previously described [26]. Titers were calculated as either the 50% inhibitory concentration (IC_50_) of mAbs or reciprocal plasma dilution (ID_50_) of plasma that caused a 50% reduction of relative light units (RLU) compared to results for the virus-treated or untreated control wells [26]. Maximum percent neutralization (%V_Max_) was defined as the maximum % neutralization observed over the range of mAb concentrations or plasma dilutions tested [12]. We also report the concentration which results in half-maximal neutralization (IC_HM_), which differs from IC_50_ when V_Max_ is less than 100% but more closely reflects the avidity of the antibody than IC_50_.

We used heatmap hierarchical clustering to categorize neutralization sensitivity of our 21 SIV virus panel, similar to the tiered categorization of neutralization sensitivity of HIV-1 viruses [27] (S2 Fig). Since several SIV strains exhibit a marked neutralization plateau effect at or below 50% neutralization [12], the heatmap was generated based on log10 values of IC80 (rather than IC_50_) titers (S3 Fig) using the web tool in the Los Alamos National Laboratory HIV sequence database with the Euclidean distance and complete clustering method. SIV strains in our panel clustered into 2 main groups of either neutralization sensitive or resistant strains.

Competition of plasma or mAb neutralization was assessed as previously described [28]. Briefly, the virus neutralization assay was performed by adding a fixed concentration (25 μg/ml) of soluble SIV proteins SIVmac239 gp140 FT, 1JO8 SIVsmE660.CP3C V1V2 and 1JO8 SIVsmE660.CR54.2A5 V1V2 [12] or irrelevant control protein (recombinant soluble HA of subtype H5 (A/Indonesia/5/2005)) to serial dilutions of plasma or mAb for 30 min prior to the addition of pseudo-typed virus. Following addition of pseudo-typed virus, the mixture was incubated for 30 min at 37°C and TZM-bl cells were added at 0.5 million cells/ml and incubated for 48 h, followed by cell lysis and measurement of luciferase activity.

### Isolation and expansion of memory B cells

Cryopreserved PBMC were thawed and stained with LIVE/DEAD Fixable Violet Dead Cell Stain (Life Technologies) as previously described [12]. Cells were washed and stained with an antibody cocktail of CD3 (clone SP34-2, BD Biosciences), CD4 (clone OKT4, BioLegend), CD8 (clone RPA-T8, BioLegend), CD14 (clone M5E2, BioLegend), CD20 (clone 2H7, BioLegend), IgA (polyclonal, Jackson ImmunoResearch), IgD (polyclonal, Dako) and IgM (clone G20-127, BD Biosciences) at room temperature in the dark for 20 mins. The stained cells were washed 3 times with PBS, re-suspended in 1 ml of PBS and passed through a 70μm cell mesh (BD Biosciences). Total class switched memory B cells (CD3-CD4-CD8-CD14-CD20+IgD-IgM-IgA-) were sorted with a modified 3-laser FACSAria cell sorter using the FACSDiva software (BD Biosciences) and flow cytometric data was subsequently analyzed using FlowJo (v9.9.5).

Bulk sorted B cells were expanded using B cell culture conditions optimized for human B cells [29]. Briefly, B cells were diluted in B cell culture medium consisting of Iscove’s modified Dulbecco’s medium (IMDM) with GlutaMAX (Thermo Fisher) supplemented with 10% heat-inactivated fetal bovine serum (FBS), 1X MycoZap Plus-PR (Lonza), 0.05 μg/ml IL-2 (Sigma-Aldrich), 0.05 μg/ml IL-21 (Thermo Fisher) and 3T3-murine (mu) CD40L feeder cells (at a density to achieve 5,000 3T3-muCD40L cells per well). Diluted cells were seeded by limiting dilution into 384-well plates at 2 cells per well and incubated at 37°C in a 5% CO2- humidified incubator for 14 days.

Single B cells were index-sorted at 1 cell/well in 384-well plates containing B cell culture medium optimized for *in vitro* expansion of rhesus B cells. Briefly, single B cells were sorted directly into wells of a 384-well plate containing IMDM with GlutaMAX, supplemented with 10% heat-inactivated fetal bovine serum (FBS), 1X MycoZap Plus-PR, 0.05 μg/ml IL-2, 0.05 ug/ml IL-21, 0.05 μg/ml IL-4 (Miltenyi Biotec), 0.08 μg/ml B cell–activating factor (BAFF) (GenScript), 2 μg/ml CpG ODN 2006 (Miltenyi Biotec) and 3T3-muCD40L feeder cells (at a density to achieve 5,000 3T3-muCD40L cells per well). Sorted cells were incubated at 37°C in a 5% CO2- humidified incubator for 14 days.

### High-throughput microneutralization assay

Single wells of B cell culture supernatant were evaluated for neutralization of SIVmac239cs23 and SIVsmE660.CR54.2A5 Env-pseudo-typed viruses using the high throughput NVITAL automated microneutralization assay as previously described [30]. Wells were selected for RT-PCR based on a 50% reduction in neutralization (calculated as reduction in relative luminescence units (RLU) compared to virus control wells after subtraction of cell control RLU).

### Immunoglobulin gene amplification and cloning

The immunoglobulin (Ig) heavy and light chain variable regions were amplified by reverse transcription polymerase chain reaction (RT-PCR) from wells that scored positive in either the SIVmac239cs23 or SIVsmE660.CR54.2A5 microneutralization assay as previously described [31]. Briefly, B cells from each well were lysed with 20 μl lysis buffer containing 0.25 μl RNase inhibitor (New England Biolabs Inc.), 0.3 μl 1M Tris pH8 (Quality Biological Inc.) and 19.45 μl diethyl pyrocarbonate (DEPC)-treated H2O. The plates containing lysed B cells were stored at -80°C. Following microneutralization screening, the frozen plates with single B-cell RNA were subsequently thawed at room temperature, and the RT reaction for neutralization-positive wells was carried out by adding 1 μl Superscript III RT (Life Technologies), 5 μl of 5x buffer, 1.25 μl 5 mM dithiothreitol (DTT), 3 μl 150 ng/ul random hexamer (Gene Link), 2 μl of 10mM dNTP mix (Qiagen), 20 U RNaseOUT recombinant RNase inhibitor (Thermo Fisher), and 0.0625 μl of Igepal (Sigma) to a 15 μl aliquot of B cell lysate. The thermocycle program for RT was 42°C for 10 min, 25°C for 10 min, 50°C for 60 min and 94°C for 5 min. The cDNA was diluted in 25μl PCR grade H_2_O before proceeding with heavy and light chain amplification with rhesus-specific primers as described previously [12,32]. Amplified PCR products were analyzed on 2% agarose gels (Embi-Tec) and positive reactions sequenced directly. Antibody variable regions were codon optimized for human cell expression, synthesized (GenScript) and cloned into rhesus CMVR expression vectors [33]. Small-scale expression and purification of full-length antibody was performed as previously described [33].

### Immunoglobulin gene family analysis

Antibody sequences were annotated in SONAR [34], using a recently published gold-standard macaque Ig database [35]. Gene nomenclature follows that used in the database, since copy-number variation in the macaque Ig locus has been insufficiently explored to confidently order genes as the standard naming convention would require.

### Antibody binding and competition assays

Enzyme-linked immunosorbent assay (ELISA) and antibody cross-competition ELISA were performed as previously described [12].

### Next generation sequencing (NGS) of naïve B cells and IgDiscover analysis

PMBC from animal IK16 were stained with anti-CD20 Alexa700-PE clone HB3 (custom conjugate), anti-IgD FITC polyclonal (Dako), anti-IgG Alexa680 clone G18-145 (custom conjugate). Live B cells were selected based on CD20^+^IgG^+^ (memory) or CD20^+^IgD^+^ (naïve) and bulk sorted into RPMI with 10% FBS and 1% Pen-Strep using a BD FACSAria II. Total RNA was extracted using RNAzol RT per the manufacturer’s guidelines (Sigma-Aldrich). Briefly, dry cell pellets were lysed in 400 μl RNAzol RT, to which 160 μl H2O was added to create an emulsion, and spun at 16,000 x g for 15 minutes at RT. The aqueous layer was removed and mixed with 5μl glycogen (4g/ml) to which 1 volume of isopropanol was added. RNA was pelleted at 16,000 x g for 15 minutes at RT, and washed twice with 75% ethanol, spinning at 16,000 x g for 2 minutes. After the final wash, the pellet was air dried for 10 minutes. RNA was resuspended in 30 μl H2O and frozen until use. cDNA libraries were generated by 5’ RACE PCR. 8 μl of RNA was hybridized to 1 μl Oligo-dT (12 μM) for 3 minutes at 72°C and cooled on ice, then added to 8.5 μl of RT master mix (First-strand buffer (1x), DTT (1.15mM), SMARTer II A oligo (0.7 μM), RNAse Out (2.3 U/μl)(Thermo Fischer Scientific), dNTP mix (0.6 mM) (Qiagen), Superscript II RT (11.4U/μl) (Thermo Fischer Scientific). The reaction was carried out at 42°C for 90 minutes, 72°C for 10 minutes, and cooled to 4°C. The cDNA was purified using AMPure XP beads (Beckman Coulter) according to manufacturer’s instructions. RACE PCR was carried out using 62.5 μl KAPA HiFi master mix (2x) (Kapa Biosystems), 0.5 μl ISPRC (5’-AAGCAGTGGTATCAACGCAGAGT-3’) (10 mM), 0.5 μl gene specific primer (10 mM), 5μl cDNA, 2.5 μl cRNA (1μg/μl) (Qiagen), and 50 μl H2O at 95°C for 3 min, cycled 30 times (95°C for 20 seconds, 57°C for 15 seconds, 72°C for 30 seconds) with a final extension at 72°C for 5 min. Reactions were subject to gel electrophoresis and bands corresponding to V region sizes were gel purified. From eluates, 15 ng of purified amplicon was used for adapter ligation PCR. The adapter reaction was carried out at 1/5 scale of the library amplification, with 4 cycles annealing at 58°C and 4 cycles annealing at 62°C using gene specific primers. Amplicons were diluted 1:10 and incorporated into a barcode ligation PCR as above in gene specific RACE PCR using unique barcode primers for each sample. The barcode ligation was carried out using thermocycler settings as in adapter ligation. Reactions were concentrated then gel purified as above. The resulting Ig variable region libraries were quantified via qPCR as and subject to NGS.

Barcoded Ig gene libraries were pooled and sequenced using an Illumina paired end MiSeq 2x300 bp read reaction (Illumina Inc). These reads were quality filtered and paired by index and sequence overlap. The resulting paired end reads were used for further downstream analysis.

A germline gene database was determined for IK16 using IgDiscover software with counsel from its developers [36] using our NGS library reads of sorted CD20^+^IgD^+^ naïve B cell Ig transcripts from IgM, IgK and IgL against a reference [37]. The output of two IgDiscover runs were crossed referenced, yielding a final germline gene database from two longitudinal timepoints retaining only sequences found in both inputs. ITS92 lineage V region genes were aligned to the resulting custom germline gene database and assigned putative unmutated germline genes.

### Production of Fabs for generating anti-idiotype antibodies

ITS103.01, ITS102.01, and ITS09 Fabs were generated by truncation of the IgG heavy chain immediately after the CH1 domain and insertion of a C-terminal AviTag/PreScission protease cleavage site/6xHIS tag (GLNDIFEAQKIEWHE/LEVLFQGPG/HHHHHH). For ITS12, a chimeric NHP-mouse antibody heavy chain was created, combining the variable and CH1 domains of the NHP ITS12 with the CH2 and CH3 of a mouse IgG1. This construct also contained C-terminal AviTag/PreScission protease/6xHIS tag.

Antibodies were expressed in Expi293F cells via transient transfection with ExpiFectamine 293 (Thermo Fisher). Supernatants were harvested 6 days post-transfection, clarified by centrifugation and 0.22 μm filter sterilized. Antibodies were purified from supernatant using Nickel Sepharose Excel Resin (Cytiva), followed by selection of correctly sized proteins on a HiLoad Superdex 200 pg 16/600 column (Cytiva).

### Immunization of mice with ITS antibodies

Ethics approval for this project was obtained from the institutional animal care and use committee (ACUC), with the protocol number 18–772. Mice were housed and cared for in accordance with local, state, federal, and institute policies in facilities accredited by the American Association for Accreditation of Laboratory Animal Care (AAALAC), under standards established in the Animal Welfare Act and the Guide for the Care and Use of Laboratory Animals.

Mice were immunized 3–4 times, with an interval of 3 weeks between prime/boosts, using 30–50 μg Fab/chimeric IgG in Sigma Adjuvant System. Serum anti-ITS humoral responses were confirmed by ELISA. Four days following the final immunization, mice were euthanized, and spleens were collected. Spleens were mechanically ruptured, and a single cell suspension was prepared using a 40 μm filter, followed by treatment with RBC Lysis Buffer (BioLegend). Cells were cryopreserved until sorting.

### Biotinylation and preparation of ITS antibody probes

Fabs or the ITS12 mouse chimera were biotinylated using BirA Biotin-protein ligase (Avidity) according to the manufacturer’s instructions. Probes were generated by combining biotinylated Fab/IgG with fluorophore-conjugated streptavidin (SA) at a 4:1 molar ratio (antibody:SA). For each ITS antibody of interest, probes were prepared using each of SA-BV650 (BioLegend) and SA-BV786 (BD), while a negative control probe (irrelevant ITS antibody isotype-matched Fab) was generated with SA-PE (BD).

### Sorting and antibody isolation from mouse B cells

Cells were sorted on a FACS Aria as described previously [38]. Briefly, single lymphocytes were gated as viable using LIVE/DEAD Fixable Violet Viability Dye (Invitrogen). B cells were then gated as being live and B220+ (PECF594; clone RA3-6B2; BD), and negative for PerCP-Cy5.5 conjugated CD3 (clone 145-2C11; BD), CD4 (clone RM4-5; BioLegend), CD8 (clone 53–6.7; BD) and F4/80 (clone BM8; BioLegend). B cells were then further gated to exclude IgD+ (BV711; clone 11–26.2a; BioLegend) and IgM+ (PE Cy7; G20-127; e-Bioscience) cells. IgG positive cells were gated on with a mixture of IgG1 (clone A85-1), IgG2a (clone R19-15), IgG2b (clone R12-3) and IgG3 (clone R40-82) (all FITC; BD). B cells of interest were then single cell sorted as negative for the negative control probe and double positive for cognate antigen. A representative gating strategy for sorting is shown in S4 Fig.

Single murine B cells were lysed, and cDNA generated as described above. Individual mouse immunoglobulin heavy, light kappa and light lambda chain genes were amplified by nested PCR using 5 μl cDNA as template. Nested PCR reactions were performed using published mouse-specific PCR primers [39]. Nested PCR products were analyzed on 1% E-Gel 96 Agarose Gels (Thermo Fisher) and samples with positive bands in both the heavy and a light chain reaction were sequenced by ACGT Inc. using the second round PCR primers.

### Expression of ITS anti-idiotype antibodies

Sequenced antibody variable regions were synthesized (GenScript) and cloned into murine heavy and light chain antibody expression vectors. Antibodies were expressed in Expi293F cells via transient transfection with ExpiFectamine 293 (Thermo Fisher), supernatants were harvested 6 days post-transfection, and clarified by centrifugation and 0.22 μm filter sterilized. Antibody-containing supernatant was used as described below or antibodies were purified from the supernatant using Protein G Sepharose 4 Fast Flow (Cytiva).

### Assessing anti-idiotype binding by ELISA

All ELISAs used total volumes of 100 μl/well (except 250 μL/well for blocking), with coating overnight in PBS at 4°C. All other incubations were performed at 37°C for 1 hour. The buffer used for blocking and reagent dilutions was Pierce Protein-Free (PBS) Blocking Buffer (Thermo Fisher). Plates were developed as described above.

The specificity of cloned anti-idiotype antibodies was assessed by indirect ELISA. 96-well ELISA plates were coated with 100 ng/well ITS mAb or a polyclonal Monkey IgG (Rockland). Unpurified supernatant was then added (diluted 1:2) in single point dilution in duplicate. Alternatively, purified protein was added at the concentrations indicated. Binding was assessed via an HRP anti-mouse secondary (Abcam; clone: SB77e, 1:5000 dilution). ELISAs were also performed coating plates with 100 ng/well anti-idiotype mAb and the ITS mAbs were titrated as the primary antibody. Binding of the ITS mAb was detected using an anti-rhesus IgG Fc-HRP (Southern Biotech; clone: SB108a).

The binding modality of the anti-idiotype antibodies was assessed via competition ELISA. ELISA plates were coated with 100 ng/well either SIVmac239 gp140 FT or SIVmac239 V1V2. Purified anti-idiotype antibodies or ITS Fabs, or an irrelevant purified antibody were titrated in the presence of a constant concentration of ITS mAb. Binding of the ITS mAb was detected using an anti-rhesus IgG Fc-HRP (Southern Biotech; clone: SB108a). The percentage blocking of ITS mAb binding was for each antibody was determined relative to the binding signal of ITS mAb alone.

### Negative-stain electron microscopy

Env protein was mixed with antibody Fab fragments at a molar ratio of 2 Fab fragments per Env protomer, followed by a 10-min incubation at 4°C. Immediately before negative staining, the samples were diluted with buffer containing 10 mM HEPES, pH 7, and 150 mM NaCl to achieve Env concentration of 0.02 mg/ml. A 4.7 μl drop of the diluted sample was applied to a glow-discharged carbon-coated electron microscopy grid and removed with blotting paper after 15 s. The grid was washed by applying consecutively 3 drops of the same buffer, followed by negative staining with three drops of 0.7% uranyl formate. Data collection was performed using a Thermo Scientific Talos F200C electron microscope equipped with a Ceta CCD camera. The pixel size was 0.25 nm, and the defocus was set at -1 μm. Particles were selected from micrographs automatically using in-house written software and subjected to 2D classification in Relion 3.1 [40].

## Results

### Broad and potent plasma neutralizing activity against SIV

Several HIV-1 bnAbs have been isolated from chronically HIV-1-infected individuals displaying broad and potent plasma neutralizing activity against HIV-1 [41–43]. However, we have rarely observed breadth of neutralizing activity against SIV that generated complete or near complete (i.e., greater than 90%) neutralization of neutralization-resistant isolates (S2 Fig) [12]. We previously reported on the isolation of a rhesus mAb clonal family (ITS90) which targets a glycan hole near the SIV gp120-gp41 interface [33]. The ITS90 clonal family of mAbs were the first SIV mAbs to fully neutralize moderately and highly neutralization-resistant SIV strains but lacked neutralization breadth in that they only neutralized SIVmac viruses.

**Fig 1:**
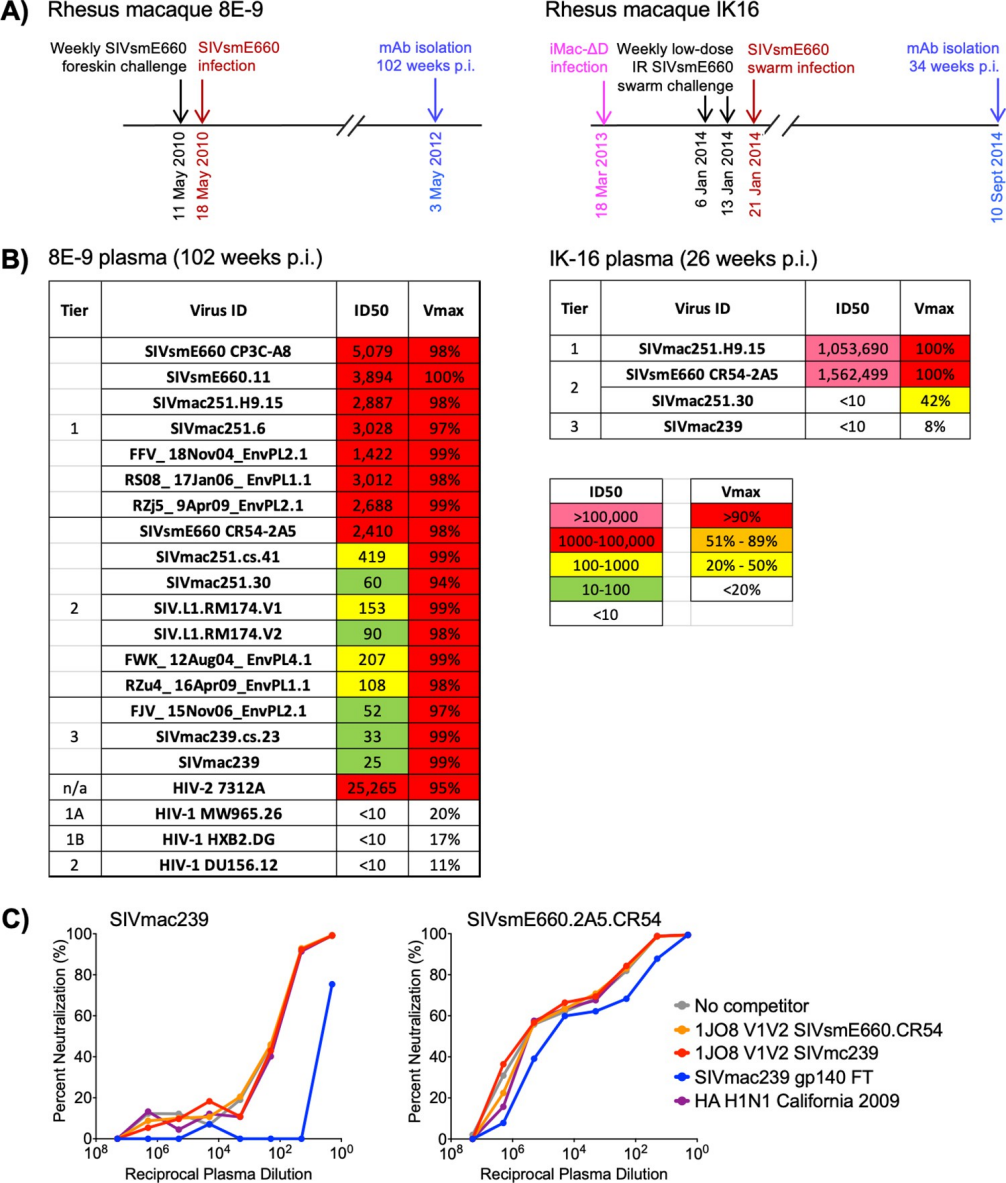
SIV-infected rhesus macaques develop broad and potent plasma neutralizing activity. **A)** Timeline showing infection history of rhesus macaques 8E-9 (left) and IK-16 (right). **B)** Neutralization profiles of plasma from 8E-9 (left) and IK16 (right) against SIV and HIV Env pseudo-typed viruses showing ID50 of plasma against each virus, and maximum % neutralization (V_Max_) observed over the range of plasma dilutions tested. **C)** Competition of 8E-9 plasma neutralization (102 weeks p.i.) of highly neutralization-resistant SIVmac239 (left) and moderately neutralization-resistant SIVsmE660.CR54.2A5 (right) by control or SIV proteins.

To identify animals with potential SIV bnAb activity, we screened plasma samples from chronic SIV-infected rhesus macaques for broad, potent, and complete neutralization activity using our panel of 21 SIV strains and HIV-2 isolate 7312A (S2 Fig). This 22-virus panel closely reflects inter-clade genetic diversity of HIV-1 [12]. Plasma from rhesus macaque 8E-9 at 102 weeks post-SIVsmE660 infection neutralized all SIV strains tested with unusually high neutralization titers against neutralization-sensitive SIV, and HIV-2 7312A (Fig 1A and 1B). More importantly, plasma from 8E-9 neutralized all strains, including neutralization-resistant SIVs (S2 Fig), at or above 94% (Fig 1B). The lack of plasma neutralizing activity against HIV-1 excluded the possibility that this unusual breadth of SIV neutralization was non-specific (Fig 1B). We also identified IK16, a rhesus macaque vaccinated with iMac-ΔD385, a non-CD4-tropic variant of SIVmac239 [20], and challenged with SIVsmE660 swarm (Figs 1A and S1), as having potent and complete neutralization of moderately neutralization-resistant SIVsmE660.CR54.2A5 albeit lacking in neutralization breadth (Fig 1B).

### Isolation of SIV bnAbs with neutralizing activity against neutralization resistant SIV

To determine whether fluorescently labeled Env protein probes could be used to sort SIV-specific B cells and isolate SIV bnAbs from animal 8E-9, we performed neutralization protein competition assays using a SIVmac239 gp140 foldon trimer (FT) to assess the ability of soluble SIV Env proteins to inhibit plasma neutralization. While FT was able to compete and deplete plasma neutralization of SIVmac239, none of the proteins tested were able to compete neutralization of SIVsmE660.CR54.2A5 (Fig 1C). Based on these finding, we determined that *in vitro* culture of unbiased class switched memory B cells followed by screening B cell culture supernatants for neutralization of SIVmac239 and SIVsmE660.CR54.2A5 would be the best approach for isolating SIV bnAbs from animal 8E-9.

**Fig 2:**
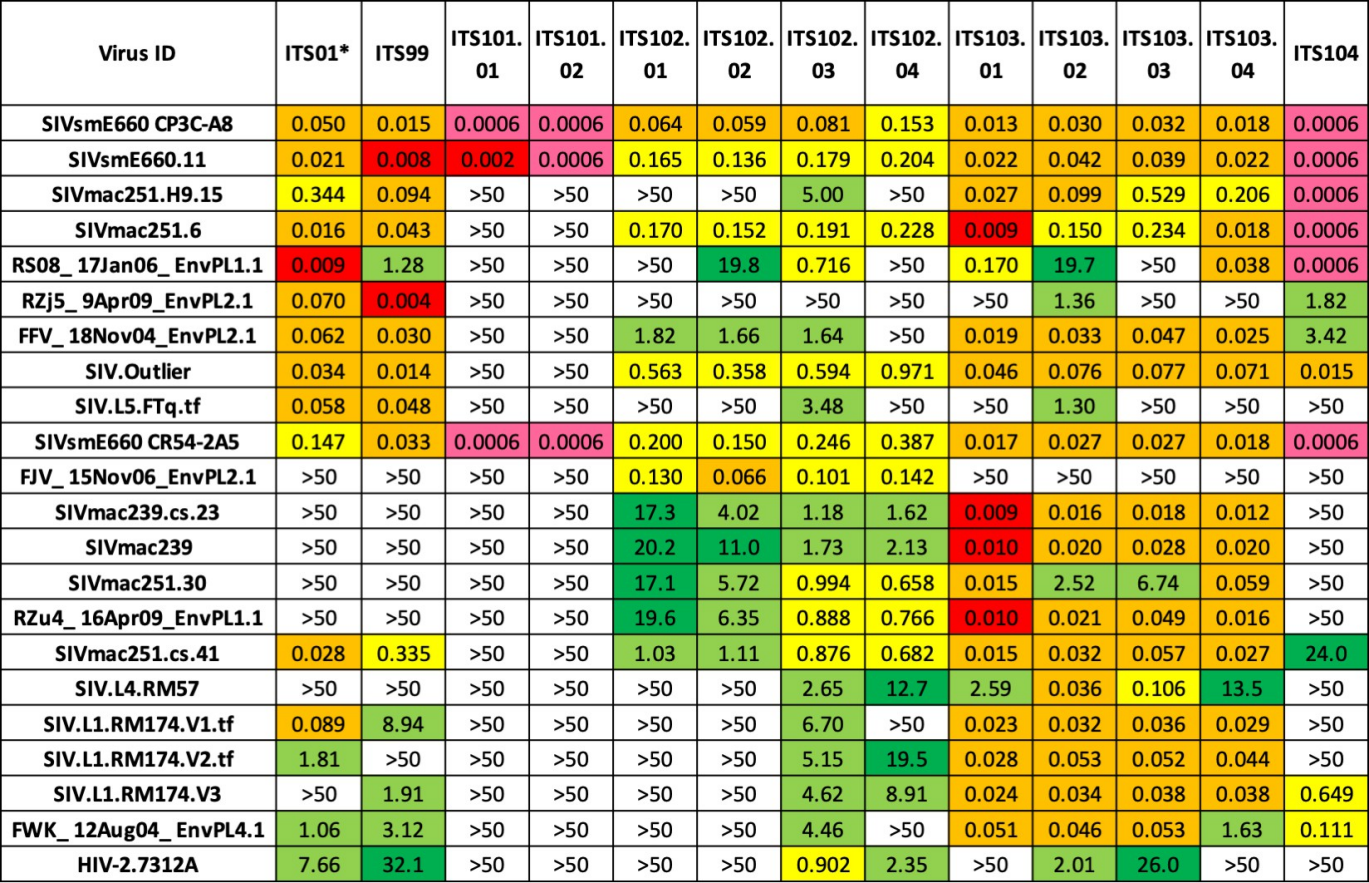
Second generation SIV mAbs target neutralization resistant SIV. Neutralization IC_50_ values of second generation SIV mAbs from animal 8E-9 against a panel of neutralization sensitive and resistant SIVs, and HIV-2 isolate 7312A. *Neutralization IC_50_ values for ITS01, a first generation SIV CD4bs neutralizing mAb, are shown for comparison.

**Fig 3:**
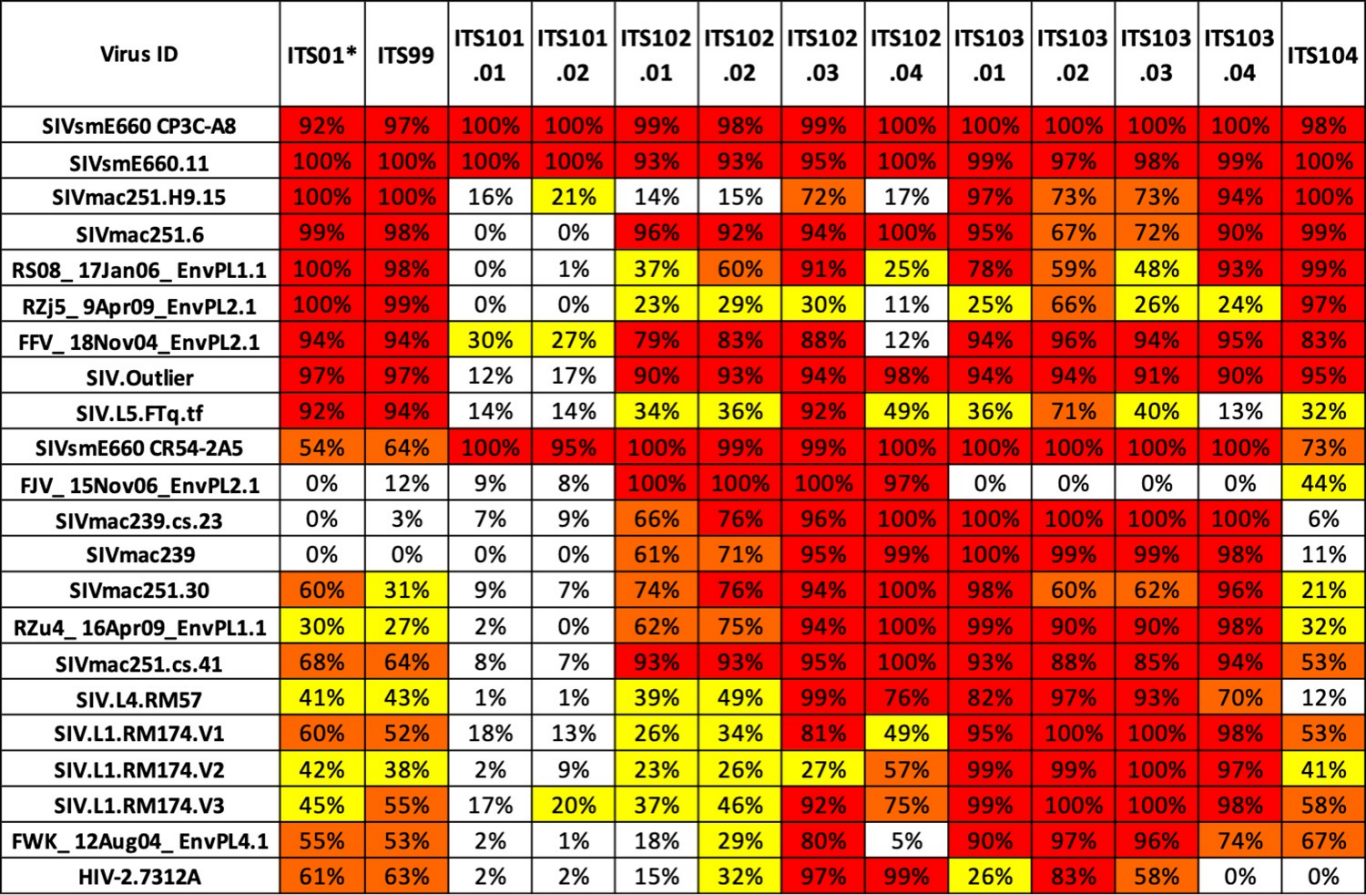
Second generation SIV mAbs mediate maximal neutralization of SIV irrespective of neutralization sensitivity. Maximum % neutralization (%V_max_) of SIV mAbs from animal 8E-9 against a panel of 21 SIV strains and HIV-2 isolate 7312A. *Neutralization %V_max_ values for ITS01, a first generation SIV CD4bs neutralizing mAb, are shown for comparison.

We isolated and cultured approximately 54,040 class switched (CD20^+^IgD^-^IgM^-^IgA^-^) memory B cells from animal 8E-9 at 102 weeks post-SIVsmE660-infection (S5 Fig) and used high-throughput microneutralization assay to screen IgG-containing culture supernatants for neutralizing activity [29]. Subsequent Ig gene cloning and mAb expression yielded 12 SIV nAbs belonging to 5 clonal families (S1 Table) (GenBank accession OL331298-OL331321). ELISA binding data revealed that all 12 SIV nAbs bound the gp120 portion of SIV Env with the ITS101 mAbs being strain specific for SIVsmE660 (S6 Fig). ITS99 and ITS104 mAbs showed some neutralization breadth but incomplete neutralization of neutralization-resistant SIV, analogous to previously isolated first generation SIV nAbs (Figs 2 and 3) [12]. Remarkably, the ITS102 and ITS103 mAbs displayed complete or near complete neutralization of most SIV strains tested, suggesting that these were bona fide SIV bnAbs capable of neutralizing even moderately and highly neutralization resistant SIV strains (Figs 2 and 3). Overall, the combination of ITS102 and ITS103 SIV bnAbs isolated from animal 8E-9 recapitulated the plasma neutralization breadth (Figs 1–3).

**Fig 4:**
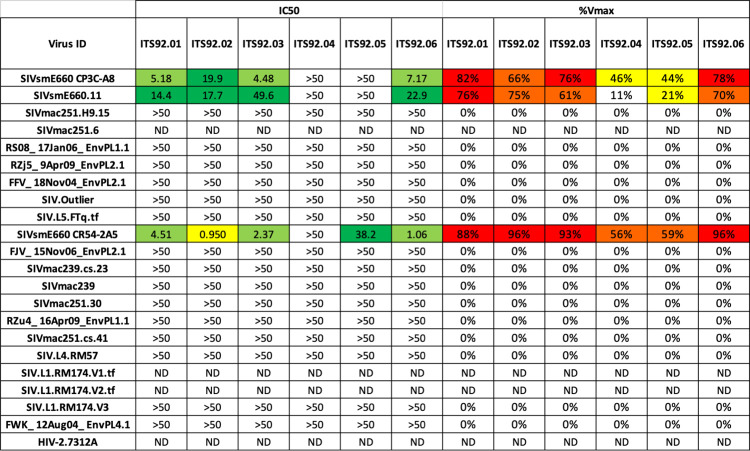
Multiple ITS92 mAbs neutralize moderately neutralization-resistant SIVsmE660.CR54.2A5. Neutralization IC_50_ and %V_max_ values of SIV mAbs from animal IK16 against a panel of 21 SIV strains and HIV-2 isolate 7312A.

We also utilized unbiased *in vitro* culture of class switched memory B cells with subsequent screening of B cell culture supernatants for neutralization of SIVsmE660.CR54.2A5 to isolate SIV nAbs from animal IK16 at necropsy when complete plasma neutralization of moderately neutralization-resistant SIVsmE660 was observed (Fig 1B). Of approximately 58,000 cells screened, we isolated 6 mAbs belonging to one clonal family (ITS92) (S2 Table) (GenBank accession numbers OL505440-OL505451) that exhibited strain-specific neutralization of SIVsmE660, including 3 nAbs (ITS92.02, ITS92.03 and ITS92.06) which achieved at least 90% neutralization of moderately neutralization-resistant SIVsmE660.CR54.2A5 (Fig 4). In contrast to plasma neutralization potency (Fig 1B), ITS92 mAbs displayed high IC_50_ values, indicating low mAb potency (Fig 4). Nonetheless, ITS92 mAbs are of particular interest given their ability to achieve near complete neutralization of moderately neutralization resistant SIVsmE660, a characteristic that distinguishes them from first generation SIV nAbs previously isolated [12].

### ITS102 and ITS103 bnAbs target the CD4bs region of SIV Env

**Fig 5:**
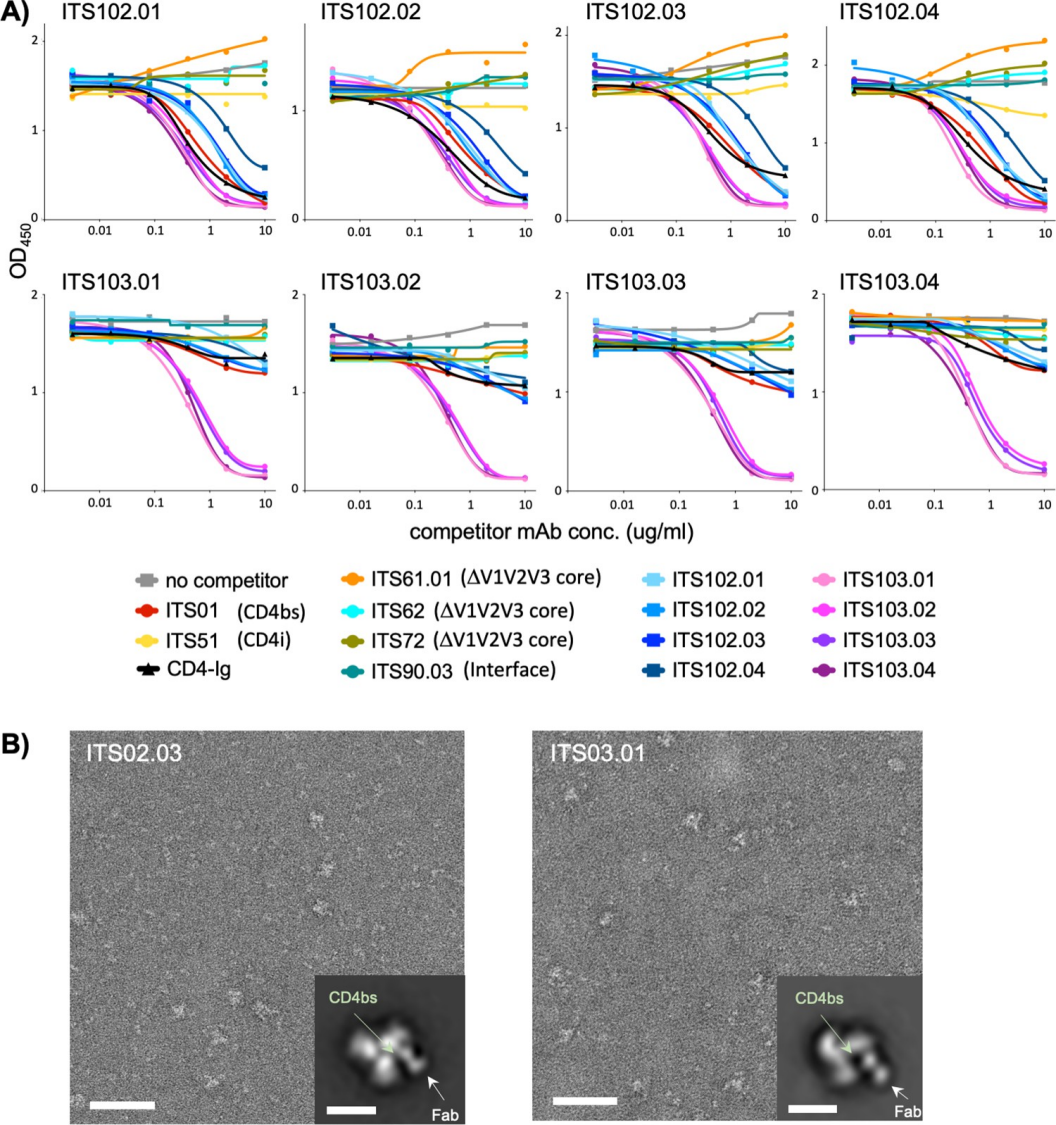
ITS102 and ITS013 bnAbs bind at or proximal to the CD4bs, respectively. **A)** ELISA competition assay showing the effects of unlabeled competitor mAbs or CD4-Ig protein on binding of biotin-labeled SIV ITS102.01-ITS102.04 and ITS103.01-ITS103.04 mAbs to SIVsmE660.CR54.2A5 gp140 foldon trimer (FT). **B)** Negative-stain electron microscopy of complexes between SIVmac239 glycoprotein and ITS02.03 (left) and ITS03.01 (right). A representative field of view with a 2D class average image showing a top view of the complex (insert) is presented for each mAb. The binding site of ITS02.03 overlaps with the CD4bs, whereas the binding site of ITS03.01 is proximal to the CD4bs. Scale bars correspond to 50 nm (fields of view) and 10 nm (2D class averages).

We sought to identify the epitope binding specificities of ITS102 and ITS103, the first reported SIV bnAbs with broad and complete or near complete neutralization of SIV. Antibody cross-competition ELISA data revealed that CD4-Ig, ITS01 (a first generation SIV CD4bs nAb displaying incomplete neutralization of neutralization-resistant SIV) [12] and ITS103 bnAbs strongly competed binding by individual ITS102 bnAbs whereas CD4-Ig and ITS01 only weakly competed binding of ITS103 bnAbs to SIV trimer (Fig 5A). This suggested that ITS102 and ITS103 bnAbs bind at and proximal to the CD4bs, respectively. To confirm this, we performed negative-stain electron microscopy (EM) on purified SIVmac239 trimer with ITS102.03 or ITS103.01 Fabs. Visual inspection of the resulting two-dimensional (2D) class averages confirmed that both ITS102.03 and ITS103.01 bind in the CD4bs region with ITS102.03 binding closer to the 3-fold trimer axis where the CD4bs is located, whereas ITS103.01 is shifted a little further away from the axis, binding proximal to the CD4bs (Fig 5B).

Since HIV-1 CD4bs bnAbs bind to residues in loop D, the CD4 binding-loop, and the V5 region [44–47], residues in these regions were mutated to generate a panel of SIVmac251.H9.15 Env pseudo-typed alanine-scanning virus mutants to evaluate the effect of specific mutations on virus neutralization and fine map the interaction of ITS102 and ITS103 bnAbs with the CD4bs region of SIV Env. The highly neutralization-sensitive strain SIVmac251.H9.15 was selected as it was expected to yield the greatest reduction in antibody neutralization. Of 40 mutants tested, 25 supported virus infectivity, and of these, only one mutation (R387A (HXB2 R367A), we follow the HIV-1 HXB2 numbering scheme from here on) affected neutralization by multiple ITS102 bnAbs (S7 Fig). The SIVmac251.H9.15 R367A mutation in the CD4 binding loop resulted in increased sensitivity to neutralization by ITS102.01, ITS102.02 and to a lesser extent ITS102.03. Of note, ITS102.03 displayed the same increased neutralization sensitivity to R367A, as well as multiple other neutralization sensitivity patterns, as observed for CD4-Ig and a first-generation SIV CD4bs nAb ITS01 [12]. By comparison, the neutralization sensitivity pattern of ITS103 bnAbs to alanine scanning mutations at the CD4bs region was distinct from that of ITS01, CD4-Ig and ITS102 bnAbs. These results are consistent with the antibody cross-competition ELISA and negative stain EM analyses (Fig 5) and demonstrate that ITS102.03 makes direct contacts similar to CD4-Ig while ITS103 bnAbs have a distinct mode of recognition from CD4-Ig or ITS102 bnAbs.

### Unusual and rare features of HIV-1 bnAbs common to SIV bnAbs

Consistent with HIV-1 bnAbs, we detected an unusually high level of somatic hypermutation (SHM) (based on nucleotide sequence divergence from putative germline genes) for ITS102 and ITS103 SIV bnAbs (S1 Table). Whereas VH and VL gene segment nucleotide mutation frequencies for first generation SIV nAbs ranged from 1%–12% [12], ITS102 and ITS103 bnAbs were 10–23% divergent from the nearest assigned germline sequences. For ITS102 bnAbs, SHM levels were higher for VL (17–19%) than VH (10–11%) genes while for ITS103 bnAbs, the opposite was observed with lower SHM levels for VL (9%) than VH (22–23%) genes.

Another key feature of HIV-1 bnAbs is the occurrence of in-frame insertions and deletions (indels), ranging from 3–33 nts, observed in 40% of HIV-1 bnAbs [48]. ITS102 and ITS103 bnAbs have indels in their VL and VH genes, respectively (S8 and S9 Figs). Sequence comparison of ITS102 and ITS013 bnAb VH genes with their nearest corresponding germline sequences revealed a 5 amino acid (aa) insertion in framework region 3 (FR3) of all ITS103 bnAbs (S9 Fig) while ITS102.01, ITS102.02 and ITS102.03 bnAbs but not ITS102.04 had a 3–4 aa deletion at the junction between FR1 and CDR H1 (S8 Fig). Interestingly, IGHV1-AFS is one of only two genes in the macaque Ig database [35] with a tryptophan at IMGT position 55, similar to human IGHV1-2, which is a key paratope residue for the VRC01-class of HIV-1 bnAbs [47–49]. Overall, IGHV1-AFS*01 conserves 8 of the 9 VRC01-class signature germline residues [50].

### Neutralization by SIV gp41-specific mAbs is strain-specific

To determine the specificity of ITS92 nAbs, we tested binding to various monomeric and trimeric soluble SIV proteins, including gp120 monomer, gp140 monomer, gp140-SOSIP trimer and scaffolded V1V2 loops (S10 Fig). Binding of ITS92.02 (and to a lesser degree ITS92.06) to gp140-SOSIP trimer but not to scaffolded V1V2 loops or any monomeric SIV proteins suggested ITS92 might target a quaternary epitope. We hypothesized that a SIV-specific quaternary-preferring mAb might facilitate stabilization of a prefusion SIV trimer complex, similar to the quaternary-specific HIV mAb PGT145 that was used to stabilize the prefusion closed conformation of soluble HIV Env trimer, BG505 SOSIP.664 [51]. Indeed, ITS92.02 Fab enabled screening of the SIV SOS-2P pre-fusion SIV-Env trimer for the E660.CR54.2A5 strain; however, the resulting cryoEM structure revealed that ITS92 was tertiary-specific rather than quaternary-specific as it bound to an epitope on SIV gp41 in the pre-fusion conformation, but which could be stabilized on a gp140 protomer (S11 Fig).

**Fig 6:**
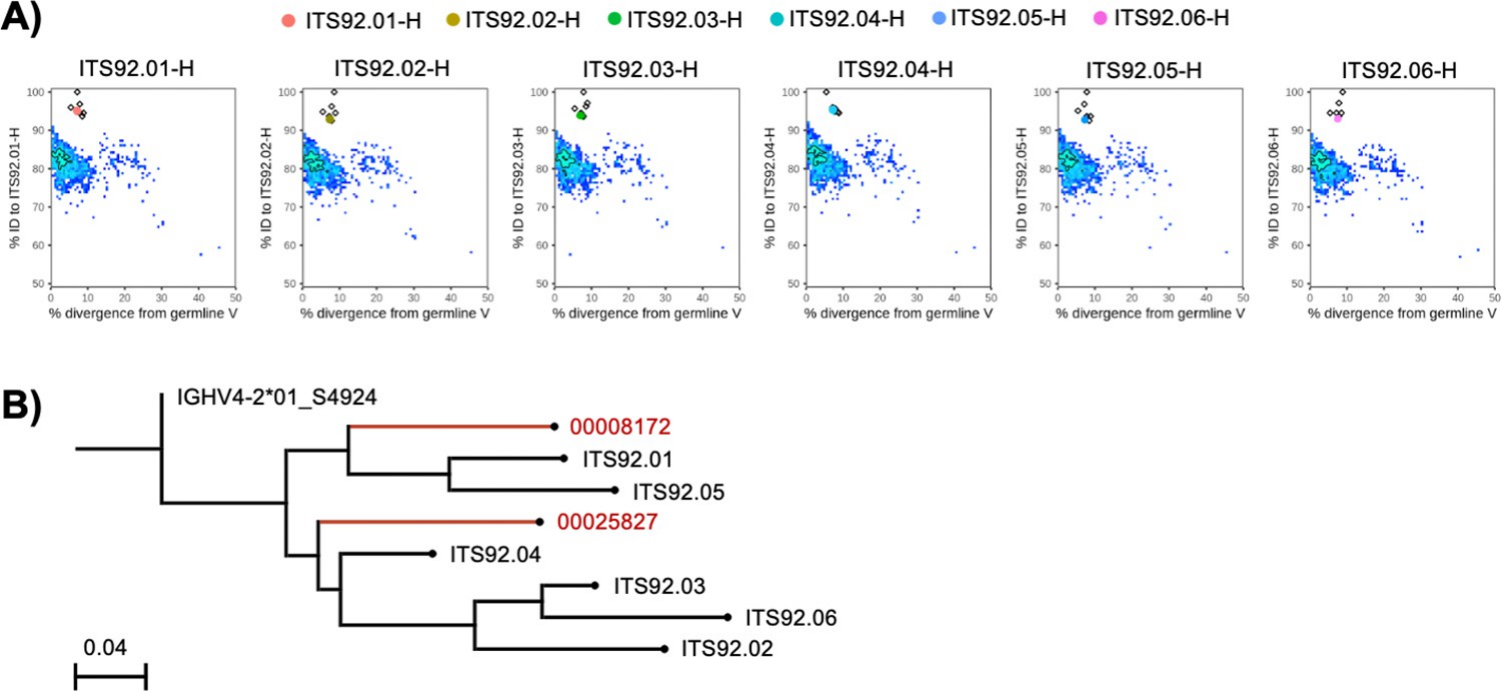
NGS analysis of ITS92 lineage mAbs. **A)** Quantitative analysis of VH region divergence from ITS92 VH (y-axis) and putative germline IGHV4-2*01_S4924 (x-axis) is displayed as pseudocolor heatmap of NGS sequence density. Sequences mapping to reference ITS92 clones (open diamonds) are denoted by colored dots (ITS92.01, Red; ITS92.02, Yellow; ITS92.03, Green; ITS92.04, Aqua; ITS92.05, Blue; ITS92.06, Magenta). **B)** Phylogeny of ITS92 VH lineage is plotted with NGS derived lineage members and the putative germline gene determined by IgDiscover.

We used next-generation sequencing (NGS) of IgM, IgK and IgL variable (V) region libraries of sorted naïve (IgD+) rhesus macaque B cells from PBMC, to create a germline gene database specific to IK16 for gamma, kappa and lambda chain V regions using the IgDiscover program [36]. The ITS92 family members mapped most closely to a novel allele termed IGHV4-2*01_S4924 and utilize the IGJH5-1*01 (S12 Fig). No D gene inference could be made given the extremely short and variable nature of this region. ITS92 VH regions diverged 7.5–12.6% from the predicted IGHV4-2*01_S4924 germline gene (S2 Table). The light chains of ITS92 mapped to a predicted novel allele IGKV1-9*01_S5333 with 4.6–8.3% divergence from germline and utilize IGKJ2*01 (S12 Fig and S2 Table). Since ITS92 nAbs utilized novel rhesus Ig alleles, we attempted to identify the ontogeny of the ITS92 lineage within NGS libraries from sorted memory (IgG^+^) B cells from four longitudinal samples spanning approximately 1 year. Only in the necropsy sample, from which ITS92 was cloned, could we identify 40 heavy and 2 light variable region sequences mapping to the ITS92 lineage (S13 Fig). The ITS92 heavy chain sequences identified by NGS from the necropsy sample grouped largely into two major branches in between ITS92 clonal members isolated from the initial B cell culture (Fig 6).

### Development of anti-idiotype antibodies to SIV bnAbs

An effective toolkit for utilizing the SIV NHP model to evaluate mAb-based interventions requires not only rhesus-derived SIV-specific mAbs but also highly specific anti-idiotypic antibodies to accurately assess pharmacokinetics, bioavailability and tissue distribution of SIV mAbs. To develop anti-idiotype antibodies for ITS102 and ITS103 SIV bnAbs as well as first-generation SIV nAbs ITS09 and ITS12 [12], mice were vaccinated with either Fabs of ITS103.01. ITS102.01 and ITS09, or a fusion protein of the ITS12 Fab with the fragment crystallizable region (Fc) of mouse antibody. Following 3–4 immunizations, single ITS-specific IgG^+^ B cells were sorted from splenocytes, and anti-idiotype antibodies cloned from sorted B cells displaying the strongest probe binding (S4 Fig).

The specificity of cloned anti-idiotype antibodies was assessed by ELISA, comparing binding to the specific ITS mAb with binding to a control polyclonal rhesus IgG. Using antibody-containing supernatant from cells transfected with anti-idiotype mAb clones, many of the anti-idiotype antibodies were able to specifically bind their cognate antibody, with up to a 50-fold increase in binding compared to the control polyclonal rhesus IgG (S14 Fig). The ability of the cloned anti-ITS103 and anti-ITS102 anti-idiotype antibodies to bind the different clonal variants of ITS103 (ITS103.01–04) and ITS102 (ITS102.01–04) was also assessed, with differences observed in the recognition of different clonal variants between anti-idiotypes (S15 Fig), suggesting the possibility that different anti-idiotype antibodies recognize distinct epitopes on the ITS mAbs.

**Fig 7:**
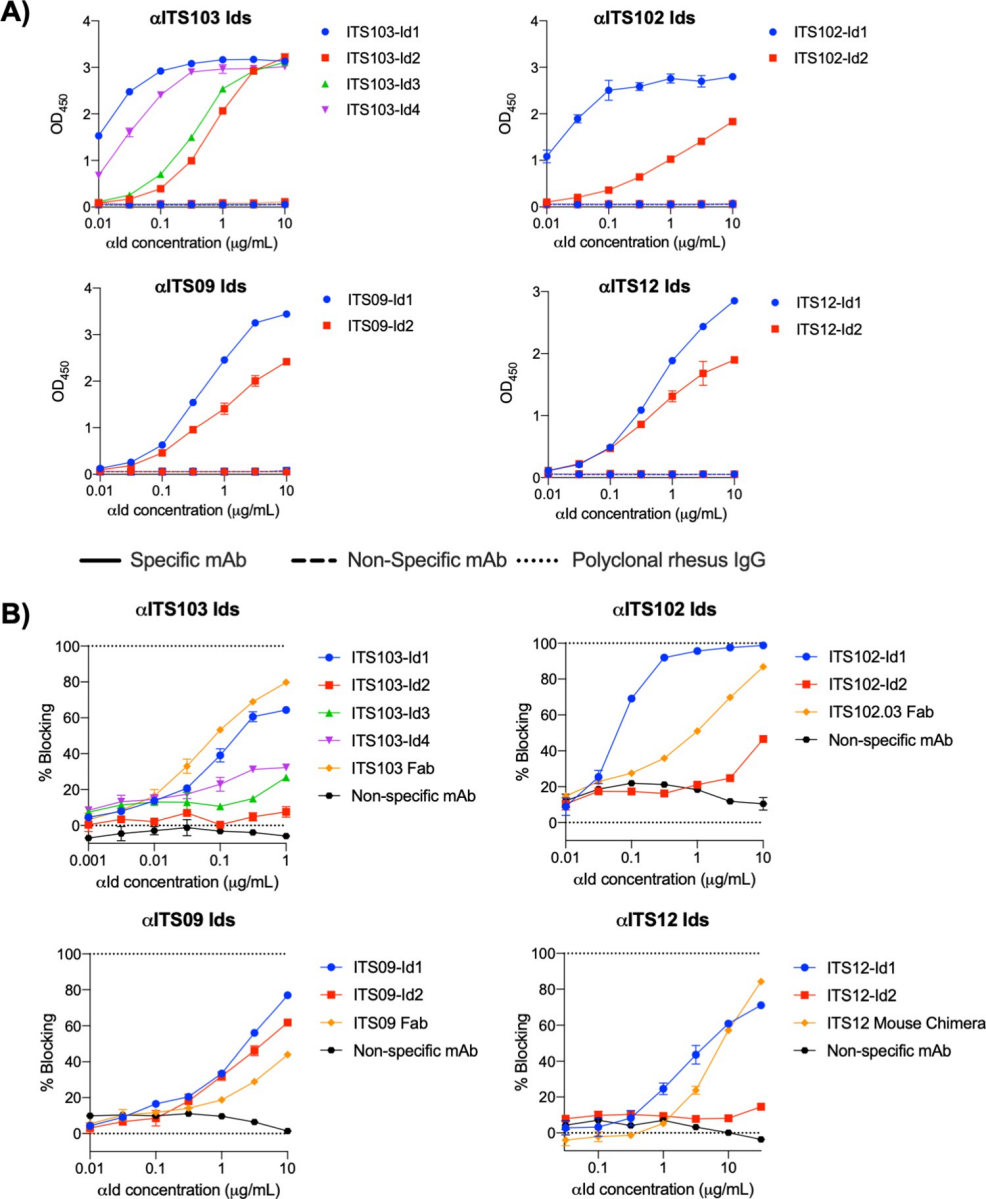
Anti-idiotype antibodies bind specifically to their cognate ITS mAbs. **A)** Binding of purified anti-ITS idiotype mAbs to ITS mAbs (specific and non-specific) and non-specific rhesus IgG by ELISA. ELISA plates were coated with 1 μg/ml ITS mAb or rhesus IgG, and anti-idiotype mAbs were titrated at the concentration indicated. **B)** Anti-idiotype antibodies able to block binding of ITS mAbs to their epitopes The ability of isolated anti-idiotype antibodies to block ITS mAb binding to their antigen was assessed by ELISA. ELISA plates were coated with Env, then the indicated amounts of anti-idiotype mAbs were titrated in the presence of 0.05 μg/ml ITS103.01 and ITS12.01, 0.1 μg/ml ITS102.01 or 0.002 μg/ml ITS09 in solution. The graphs here express percent blocking (i.e., the percentage reduction in binding when the anti-idiotype antibodies were added).

From this initial screen, anti-Id mAbs were selected for larger scale expression and purification based upon their strength of binding and binding pattern to the different clonal variants of the ITS mAbs. Purified anti-idiotype mAbs were screened for their ability to bind to the matched ITS-mAb, a non-cognate ITS mAb of the same isotype, and the control rhesus IgG. The selected anti-idiotype antibodies all displayed negligible binding to the non-cognate ITS mAb and control rhesus IgG, but strong binding to the matched mAb (Fig 7). When coated onto an ELISA plate, the anti-Id mAbs were able to detect the binding of a large dynamic range of ITS mAb (S16 Fig) Competition ELISA was used to assess whether the selected anti-idiotype antibodies were able to block binding of their mAb to antigen. Most of the selected anti-idiotype mAbs were able to block binding of the ITS-mAb to antigen, indicating the anti-idiotype likely binds the ITS antibody’s paratope (Fig 7). However, in most cases a large amount of anti-idiotype mAb was needed to fully block the binding of a relatively small amount of ITS-mAb, perhaps indicating the affinity of these anti-idiotype antibodies for the ITS mAb to be lower than that of the ITS mAb for Env.

## Discussion

Although SIV closely recapitulates HIV-1 infection, pathogenesis, and genetic diversity [52–55], the SIV NHP model for evaluating HIV-1 mAb-based interventions is under-utilized, largely owing to the high neutralization resistance of challenge strains [14,18,56] and the paucity of broadly reactive SIV Env-specific mAbs analogous to HIV-1 bnAbs. We previously reported on the isolation of more than 70 rhesus-derived SIV Env-specific mAbs which, while broadly cross-reactive, displayed incomplete neutralization of moderately and highly neutralization-resistant SIV [12]. More recently, we isolated a clonal family of SIV mAbs, reactive with the SIVmac CD4 binding site, capable of fully neutralizing moderately and highly neutralization resistant SIVmac isolates but neutralization breadth was restricted to SIV strains lacking a glycan at residue 238 [33]. As such, SHIV models in NHPs have been employed extensively to address questions of the role neutralizing antibodies in vivo using anti-HIV-1 bnAbs to evaluate treatment and prevention strategies [57–60]. In this study we sought to isolate and characterize rhesus-derived SIV Env-specific bnAbs that, in contrast to previous nAbs, could fully neutralize moderately and highly neutralization-resistant SIV strains as tools for evaluating bnAb-based prevention and treatment strategies in SIV-based NHP model.

Systematic screening of HIV-infected plasma samples for breadth of neutralization activity has led to the isolation of numerous HIV-1 bnAbs with promising potential for widespread clinical use [6,61,62]. In this study, we identified 2 animals, 8E-9 and IK16, with full neutralizing activity (i.e., >90% neutralization) against moderately and highly neutralization-resistant SIV strains. Notably, of nearly 100 chronically SIV-infected rhesus macaques tested, only 8E-9 and IK16, as well as an animal from which we previously isolated ITS90 lineage mAbs targeting the gp120-gp41 interface of SIV [33], had potent plasma neutralizing activity that achieved greater 90% neutralization against moderately or highly neutralization-resistant SIV strains. This is consistent with the observation that while serum cross-reactive neutralizing activity in chronically HIV-infected individuals is not uncommon, individuals with broad and potent serum neutralizing activity, i.e., elite controllers, are rare, comprising only 1% of HIV-infected individuals [63–67].

By focusing on an NHP with broad and potent plasma neutralizing activity, it is not surprising that we readily isolated SIV bnAbs that fully neutralized most moderately and highly neutralization resistant SIV strains. Two clonal families of SIV mAbs isolated from 8E-9 were of particular interest: ITS102 and ITS103, each with 4 clonal members. ITS102 and ITS103 were found to target epitopes overlapping or proximal to the CD4bs region, respectively. Importantly, all ITS102 and ITS103 mAbs neutralized one or more moderately or highly neutralization resistant SIV strains with ITS103 mAbs displaying greater neutralization breadth and potency compared to ITS102 mAbs. Similar to HIV-1 bnAbs which generally have gaps in their neutralization breadth coverage [3], none of the ITS102 or ITS103 mAbs provided coverage against all SIV strains tested. Nonetheless, ITS102.03 and ITS103.01 stood out for their ability to fully neutralize most SIV strains tested and, more importantly, the non-overlapping neutralization profiles of ITS102.03 and ITS103.01 suggest that the combination of these bnAbs may provide greater SIV neutralization breadth coverage. Indeed, a key concern for HIV-1 bnAb-based interventions is selecting the ideal combination of bnAbs to achieve optimal neutralization coverage. Therefore, ITS102 and ITS103 mAbs should be highly useful in evaluating combination bnAb-based vaccines or therapies in the SIV NHP model.

In addition to isolating SIV bnAbs targeting the CD4bs, we also isolated a clonal family of 6 nAbs targeting SIV gp41. While the ITS92 mAbs we report herein are not the first reported SIV gp41-specific nAbs [68], and display varying levels of strain-specific neutralizing activity against SIVsmE660 only, several ITS92 nAbs are notable for their ability to achieve near complete neutralization of SIVsmE660.CR54.2A5, a transmitted founder Env clone with significant resistance to neutralization by SIV mAb or plasma antibodies [56].

Finally, given the potential significance of SIV CD4bs region-specific ITS102 and ITS103 bnAbs for testing bnAb-based interventions for HIV-1, we isolated several anti-idiotype mAbs with specificity for ITS102 and ITS103. We also isolated anti-idiotype mAbs specific for first-generation SIV V2-specific nAbs ITS09 and ITS12 [12] which have been of particular interest for NHP mAb studies [69,70] owing to the strong correlation between IgG Env V1V2-binding antibody levels and the modest efficacy observed in the RV144 Phase III vaccine trial [71]. The isolation of multiple anti-idiotypes raised against SIV-specific mAbs that we describe here will enable the development of assays to monitor serum pharmacokinetics and quantitate epitope-specific immune responses to these SIV mAbs.

In summary, we have isolated the first SIV CD4bs region-specific bnAbs, SIV gp41-specific nAbs, and anti-idiotype antibodies targeting multiple SIV-specific mAbs, thereby expanding the toolkit of reagents which can be used in the SIV NHP model to rigorously evaluate mAb-based interventions to treat or prevent SIV/HIV-1 infection.

## Supporting information

S1 Table Immunogenetics of SIV mAbs isolated from rhesus macaque 8E-9. (TIF)

S2 Table Immunogenetics of SIV mAbs isolated from rhesus macaque IK16. (TIF)

S1 Fig Viral RNA & neutralization titers for iMac-ΔD rhesus macaque IK16. **A)** Longitudinal quantification of SIV RNA and **B)** plasma neutralization titers pre- and post- intra-rectal SIVsmE660 challenges.
(TIF)

S2 Fig Categorization of SIV strains based on sensitivity to mAb neutralization. Heatmap showing neutralization-sensitive and -resistant SIV strains based on hierarchical clustering on log10 values of IC80 neutralization titers by SIV mAbs targeting various epitopes. Red asterisk denotes those strains which exhibit a marked neutralization plateau effect irrespective of antibody specificity.
(TIF)

S3 Fig Neutralization IC80 values of second generation SIV mAbs. Neutralization IC80 titers of SIV mAbs against SIV strains used for heatmap hierarchical clustering to categorize neutralization sensitivity of our 21 SIV virus panel.
(TIF)

S4 Fig Sorting strategy to isolate single ITS103-specific B cells. Gating strategy for isolation of memory B cells from spleens of mice immunized with ITS103 mAb showing sequential gating of lymphocytes / singlets / Live / CD3-CD4-CD8-F4/80- / CD20+ / IgM- / IgD- / IgA+ cells. ITS103 (positive) and ITS102 (negative) probes were used to sort for ITS103-specific B cells.
(TIF)

S5 Fig Isolation of switch memory B cells. Representative gating strategy for isolating rhesus macaque switch memory B cells from animal 8E-9 showing sequential gating of lymphocytes / singlets / Live / CD3-CD4-CD8-CD14- / CD20+ / IgM- / IgD- / IgA- cells.
(TIF)

S6 Fig SIV bnAbs bind gp120 portion of SIV Env. ELISA binding of second-generation SIV mAbs to gp140 foldon trimer (FT) (top) and V1V2V3-deleted gp120 core (bottom) proteins derived from SIVmac239 (left) and SIVsmE660 (right).
(TIF)

S7 Fig Neutralization by ITS102.03 and CD4-Ig is similarly affected by CD4bs residue mutations. A panel of SIVmac251.H9.15 alanine scanning Env pseudo-typed virus mutants were used to map the neutralizing activity of SIV bnAbs. IC50 WT / IC50 mutant ratios < 0.5 and > 2 indicate increased sensitivity (red) or resistance (blue), respectively relative to wild-type SIVmac251.H9.15. Viruses marked with an asterisk (*) indicates viruses not tested due to low/absent infection.
(TIF)

S8 Fig Sequence alignments of ITS102 SIV bnAbs. Alignment of the deduced amino acid sequences of the variable regions of ITS102 bnAbs to predicted germline IGHV and IGLV genes. Framework (FR) and CDRs are indicated above each sequence alignment.
(TIF)

S9 Fig Sequence alignments of ITS103 SIV bnAbs. Alignment of the deduced amino acid sequences of the variable regions of ITS103 bnAbs to predicted germline IGHV and IGLV genes. Framework (FR) and CDRs are indicated above each sequence alignment.
(TIF)

S10 Fig ITS92 family mAbs selectively bind native SIV trimer. ITS92 family antibodies were tested for binding to various SIV proteins. Binding avidity is expressed as follows: ++++, OD450 ≥ 3.0 and EC50 ≤ 0.1 μg/ml; +++, OD450 ≥ 3.0 and EC50 > 0.1 μg/ml; ++, 1.0 ≤ OD450 < 3.0; +, 0.2 ≤ OD450 < 1.0.
(TIF)

S11 Fig The ITS92.02 epitope is dependent on the prefusion tertiary conformation of gp41. A) ITS92.02 bound to a SIV E660.CR54.2A5 SOS-2P dimer is shown in two orientations in cartoon format. B) The HIV-1 BG505 SOSIP trimer (PDB ID 4TVP) is shown next to an overlay of one HIV-1 prefusion gp41 chain (pink) with a gp41 chain from the ITS92.02-bound dimer (grey). A red box highlights the well-aligned ITS92.02 epitope region. C) Postfusion gp41 from SIV mac239 (PDB ID 1QBZ) is shown next to a single gp41 chain (light cyan) aligned with a chain from the ITS92.02-bound dimer (grey). D) One protomer of the SIV E660.CR54.2A5 dimer is shown next to the aligned SIV mac239 postfusion conformation. E) The gp41 chain of prefusion and postfusion gp41 conformations are shown with a 5 Å footprint of the ITS92.02 epitope is shown in red for prefusion and blue for postfusion. Epitope residues within the 638–652 helical range align well while residues within the 608–614 loop extend distantly away.
(TIF)

S12 Fig Sequence alignments of ITS92 SIV nAbs. Alignments of the deduced amino acid sequences of the variable regions of ITS92 nAbs to predicted germline IGHV and IGKV genes. Framework (FR) and CDRs are indicated above each sequence alignment.
(TIF)

S13 Fig NGS identified 40 VH and 2 VK genes with identity to ITS92 nAbs. NGS data was probed for VH and VK regions with homology to ITS92 heavy and light chains. Alignments of VH (top) and VK (bottom) genes identified by NGS (red) to ITS92 and putative germline genes are displayed.
(TIF)

S14 Fig Screening of cloned anti-idiotype antibodies for binding specificity. Binding of supernatant from transiently transfected 293F cells was assessed towards the specific ITS antibody (ITS103.01, ITS102.01, ITS09 or ITS12.01) and also to a pool of non-specific rhesus IgG by ELISA. ELISA plates were coated with 1 μg/mL ITS mAb, then cell culture supernatant from cells expressing anti-idiotype mAbs was used at a 1:1 dilution with buffer as the primary antibody. Shown here is the ratio of binding to this positive antibody and the negative rhesus IgG. X-axis labels indicate the anti-idiotype clone name, while labels above indicate names of the selected anti-Ids.
(TIF)

S15 Fig Binding of anti-idiotype antibodies to individual SIV antibodies. Binding of A) anti-ITS103 and B) anti-ITS102 mouse mAbs to different ITS103 (ITS103.01, ITS103.02, ITS103.03 and ITS103.04) and ITS102 (ITS102.01, ITS102.02, ITS102.03 and ITS102.04) clonal variants measured by ELISA. ELISA plates were coated with 1 μg/mL ITS clonal variants, then cell culture supernatant from cells expressing anti-idiotype mAbs was used at a 1:1 dilution with buffer as the primary antibody.
(TIF)

S16 Fig Titration of ITS mAbs against anti-idiotype antibodies. Binding of ITS mAbs to anti-idiotype mAbs was measured by ELISA. ELISA plates were coated with 1 μg/mL anti-idiotype mAb, and ITS mAbs were titrated at the concentration indicated.
(TIF)
