# Supplementary figures and images for: Broad coverage of neutralization-resistant SIV strains by second-generation SIV-specific antibodies targeting the region involved in binding CD4
Source: PLoS Pathog. 2022 Jun 16;18(6):e1010574. doi: 10.1371/journal.ppat.1010574 (PMC9242510; doi:10.1371/journal.ppat.1010574)

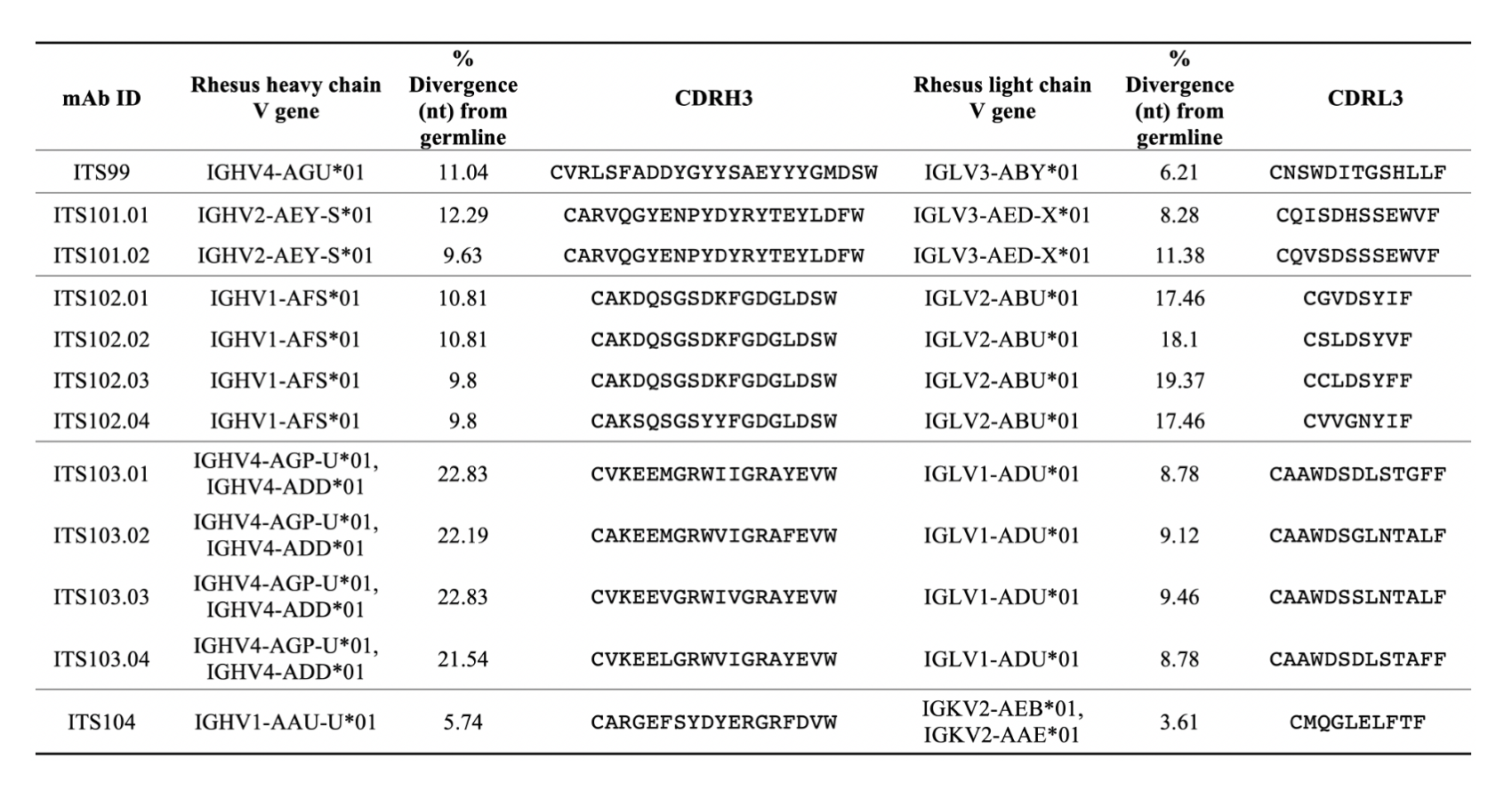

Supplement: S1 Table — (TIF) [file ppat.1010574.s001.tif]

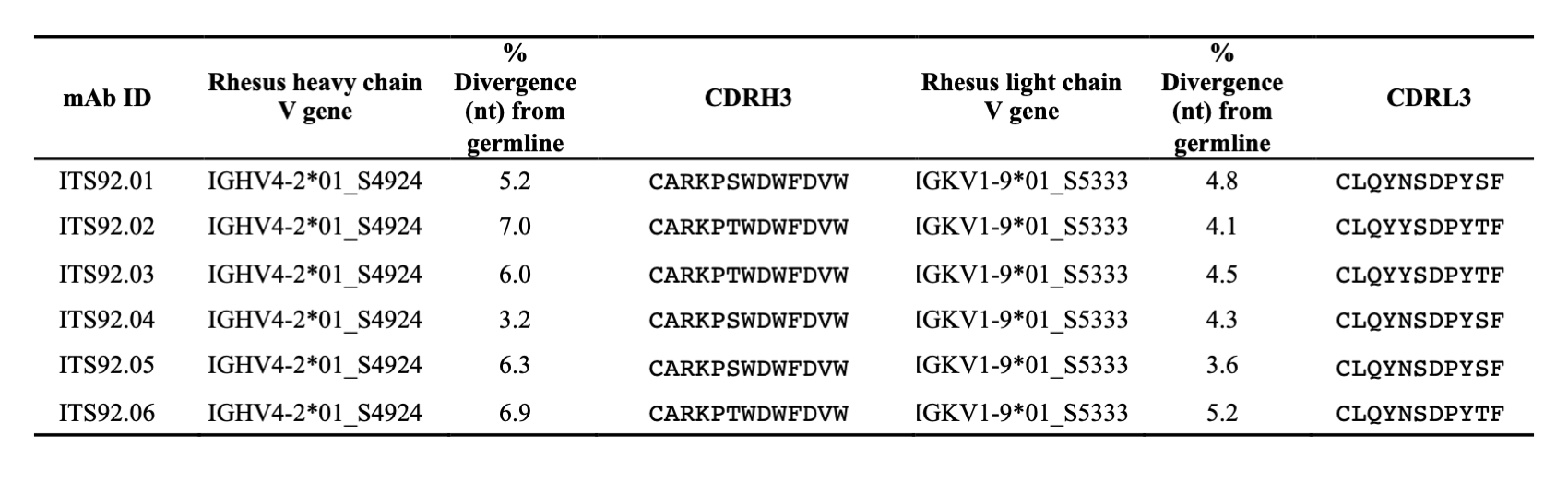

Supplement: S2 Table — (TIF) [file ppat.1010574.s002.tif]

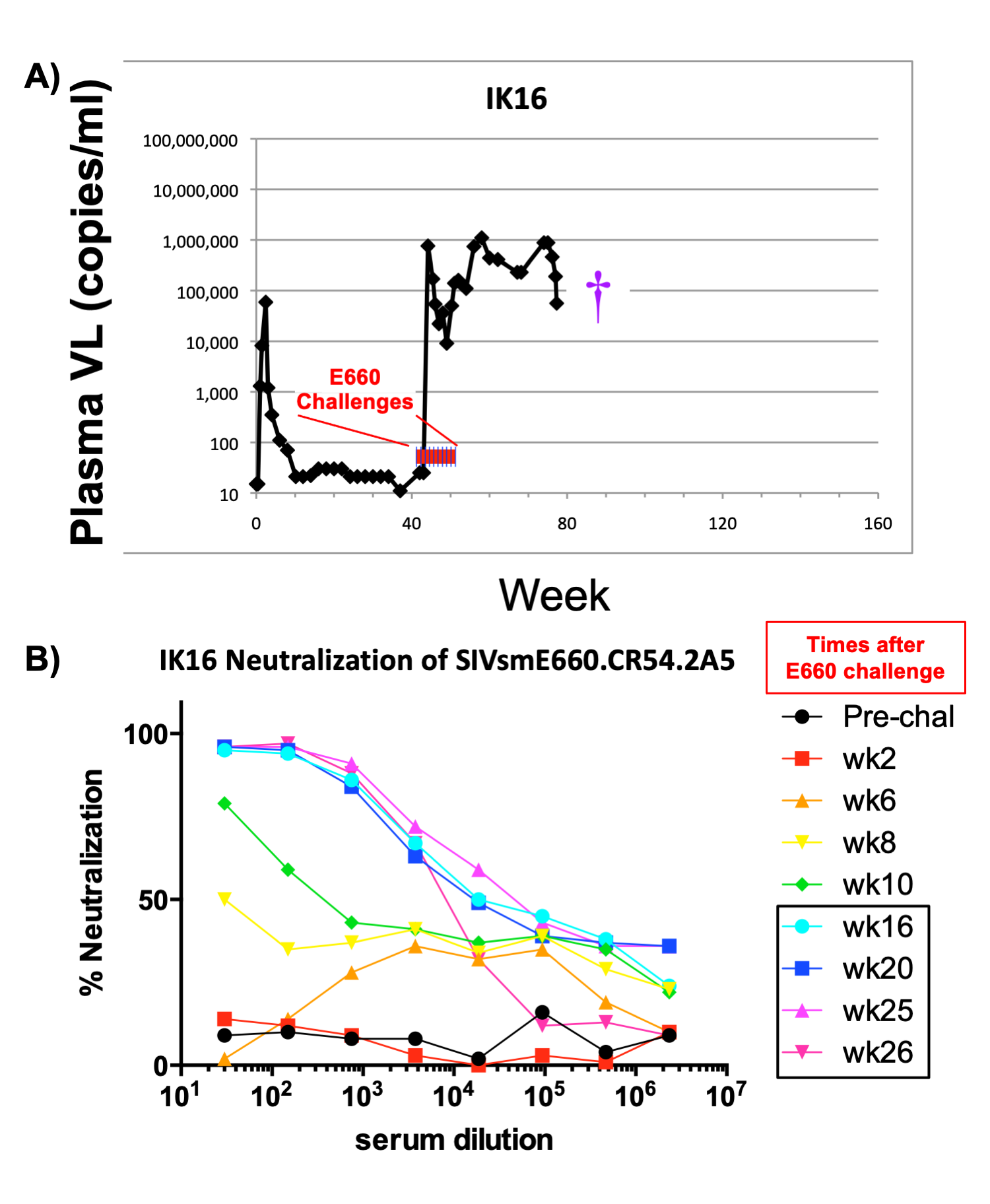

Supplement: S1 Fig — A) Longitudinal quantification of SIV RNA and B) plasma neutralization titers pre- and post- intra-rectal SIVsmE660 challenges. (TIF) [file ppat.1010574.s003.tif]

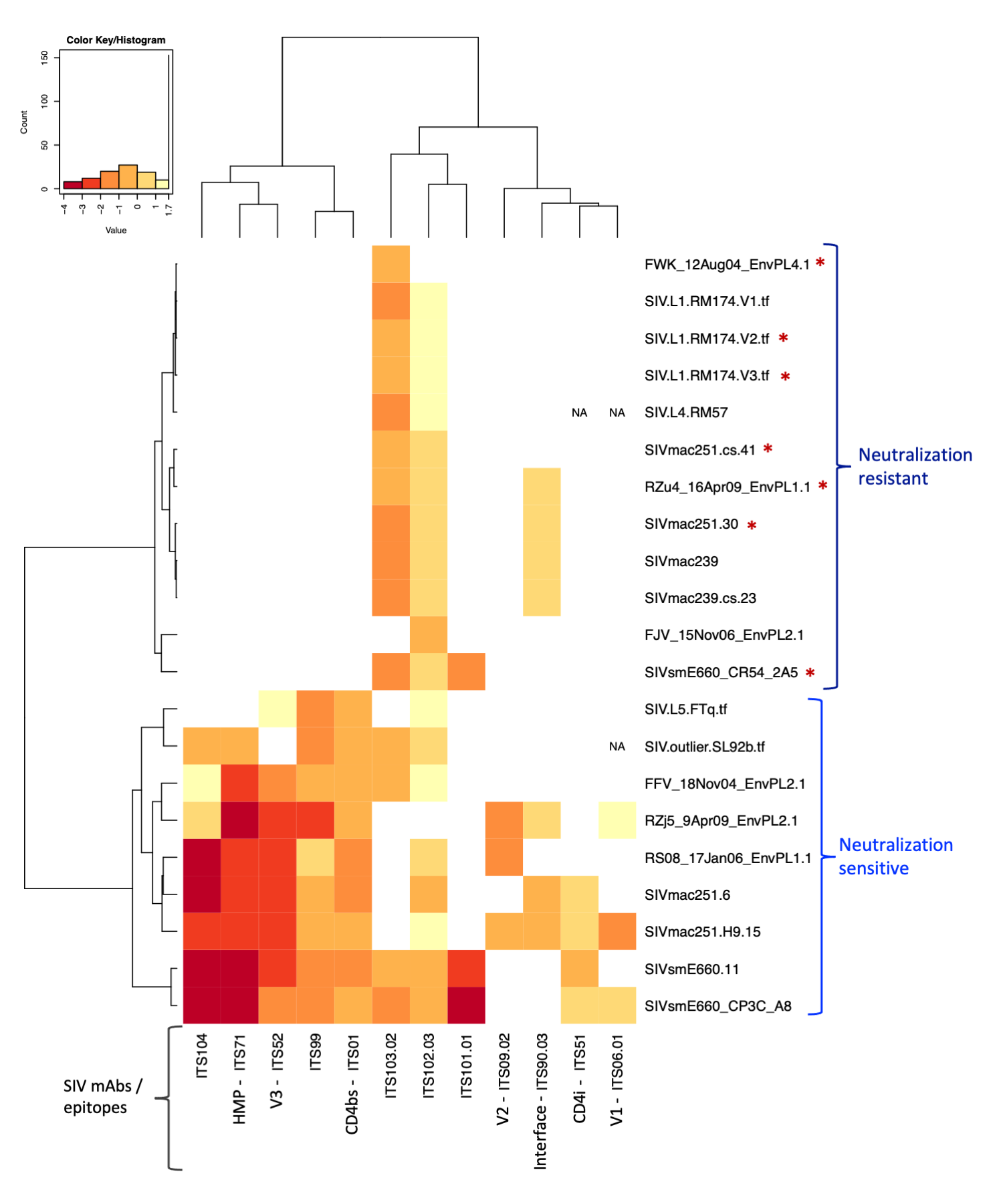

Supplement: S2 Fig — Heatmap showing neutralization-sensitive and -resistant SIV strains based on hierarchical clustering on log10 values of IC80 neutralization titers by SIV mAbs targeting various epitopes. Red asterisk denotes those strains which exhibit a marked neutralization plateau effect irrespective of antibody specificity. (TIF) [file ppat.1010574.s004.tif]

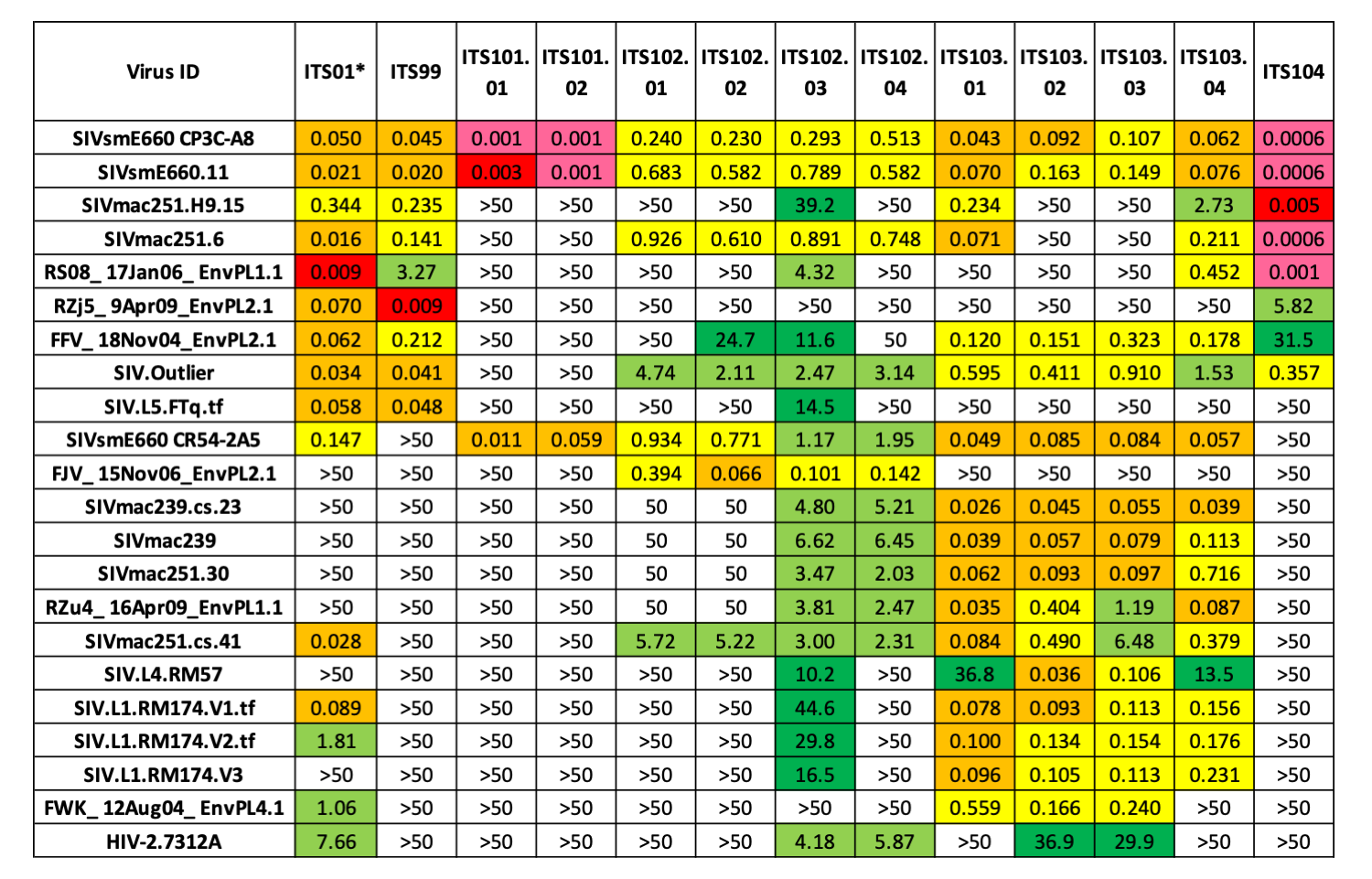

Supplement: S3 Fig — Neutralization IC80 titers of SIV mAbs against SIV strains used for heatmap hierarchical clustering to categorize neutralization sensitivity of our 21 SIV virus panel. (TIF) [file ppat.1010574.s005.tif]

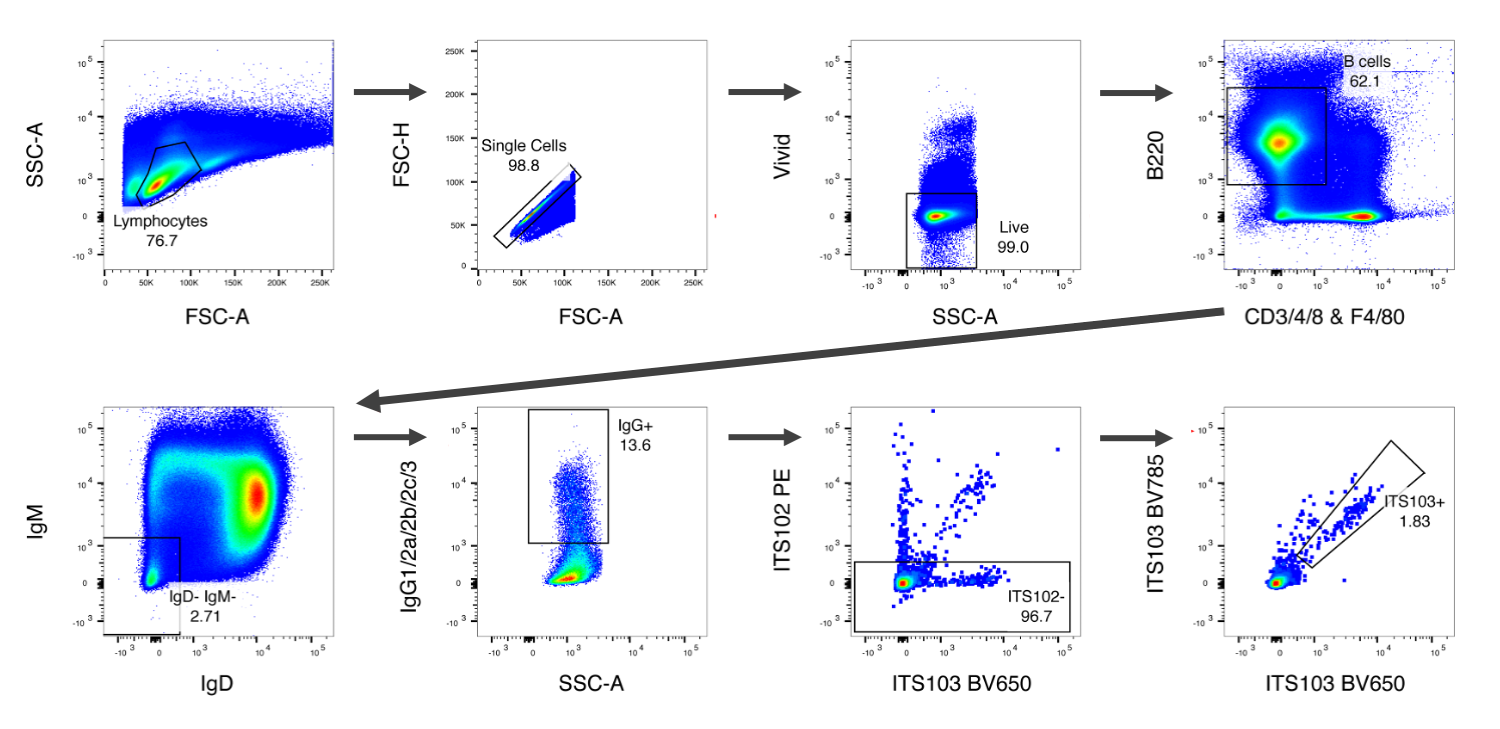

Supplement: S4 Fig — Gating strategy for isolation of memory B cells from spleens of mice immunized with ITS103 mAb showing sequential gating of lymphocytes / singlets / Live / CD3-CD4-CD8-F4/80- / CD20+ / IgM- / IgD- / IgA+ cells. ITS103 (positive) and ITS102 (negative) probes were used to sort for ITS103-specific B cells. (TIF) [file ppat.1010574.s006.tif]

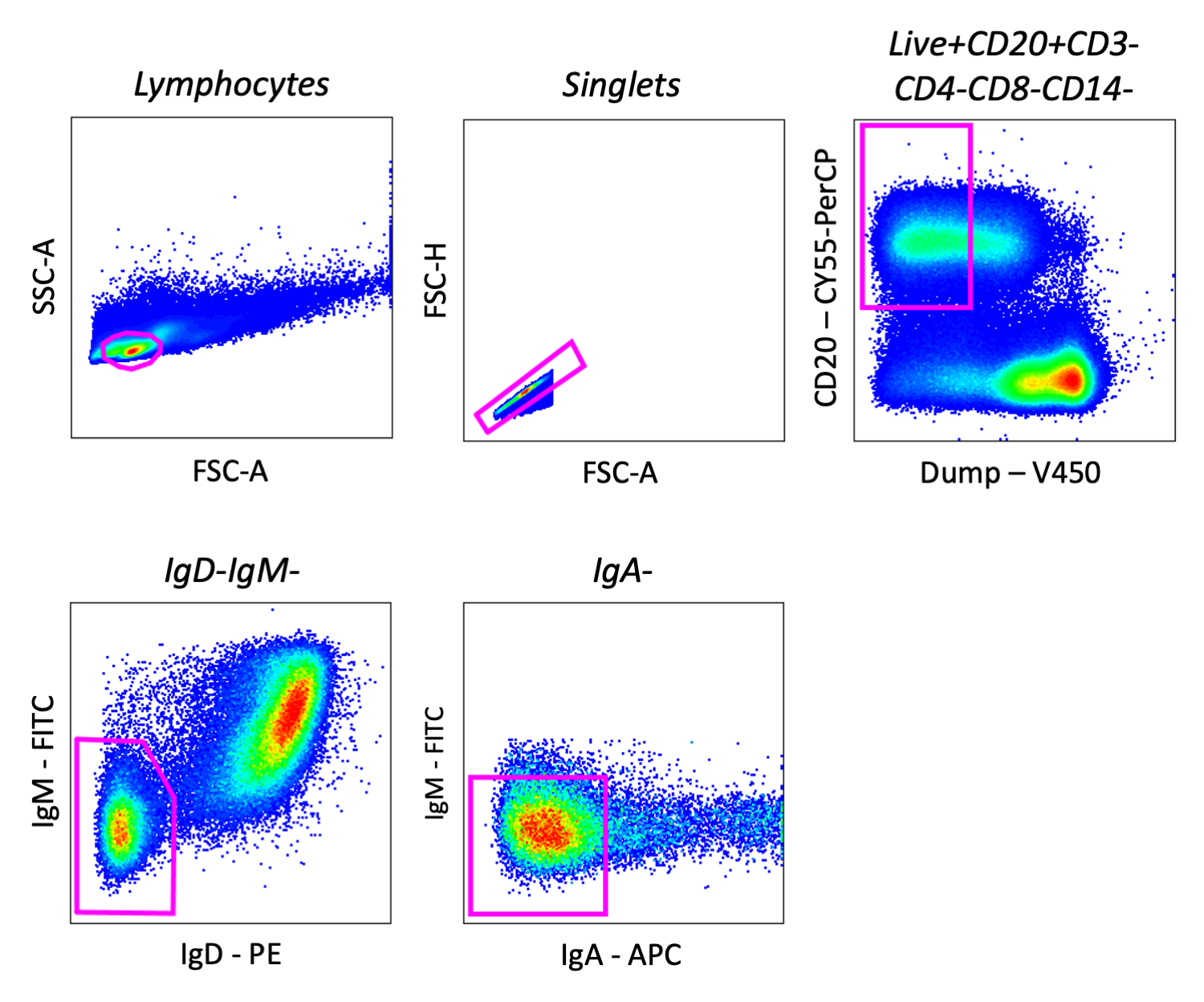

Supplement: S5 Fig — Representative gating strategy for isolating rhesus macaque switch memory B cells from animal 8E-9 showing sequential gating of lymphocytes / singlets / Live / CD3-CD4-CD8-CD14- / CD20+ / IgM- / IgD- / IgA- cells. (TIF) [file ppat.1010574.s007.tif]

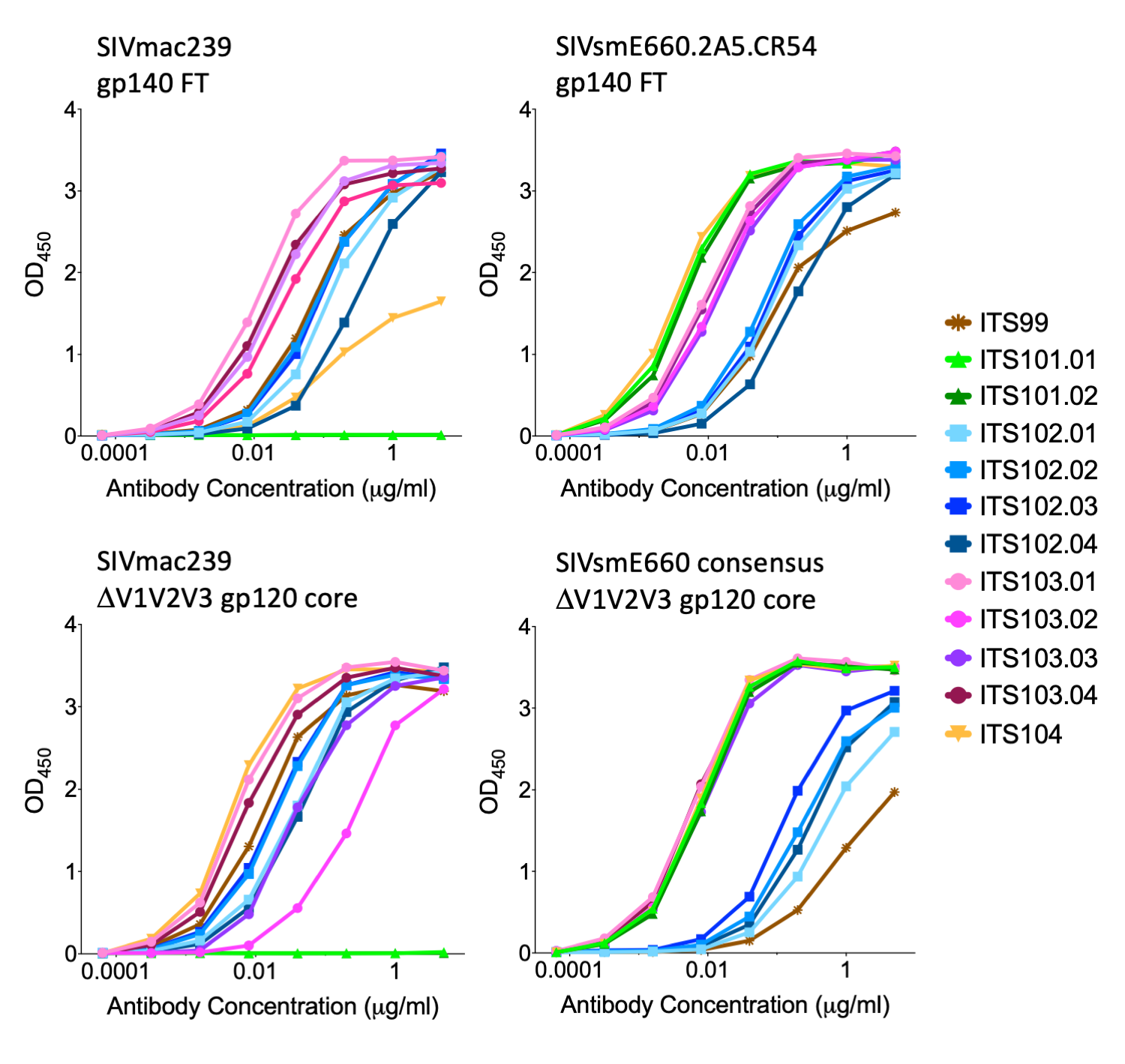

Supplement: S6 Fig — ELISA binding of second-generation SIV mAbs to gp140 foldon trimer (FT) (top) and V1V2V3-deleted gp120 core (bottom) proteins derived from SIVmac239 (left) and SIVsmE660 (right). (TIF) [file ppat.1010574.s008.tif]

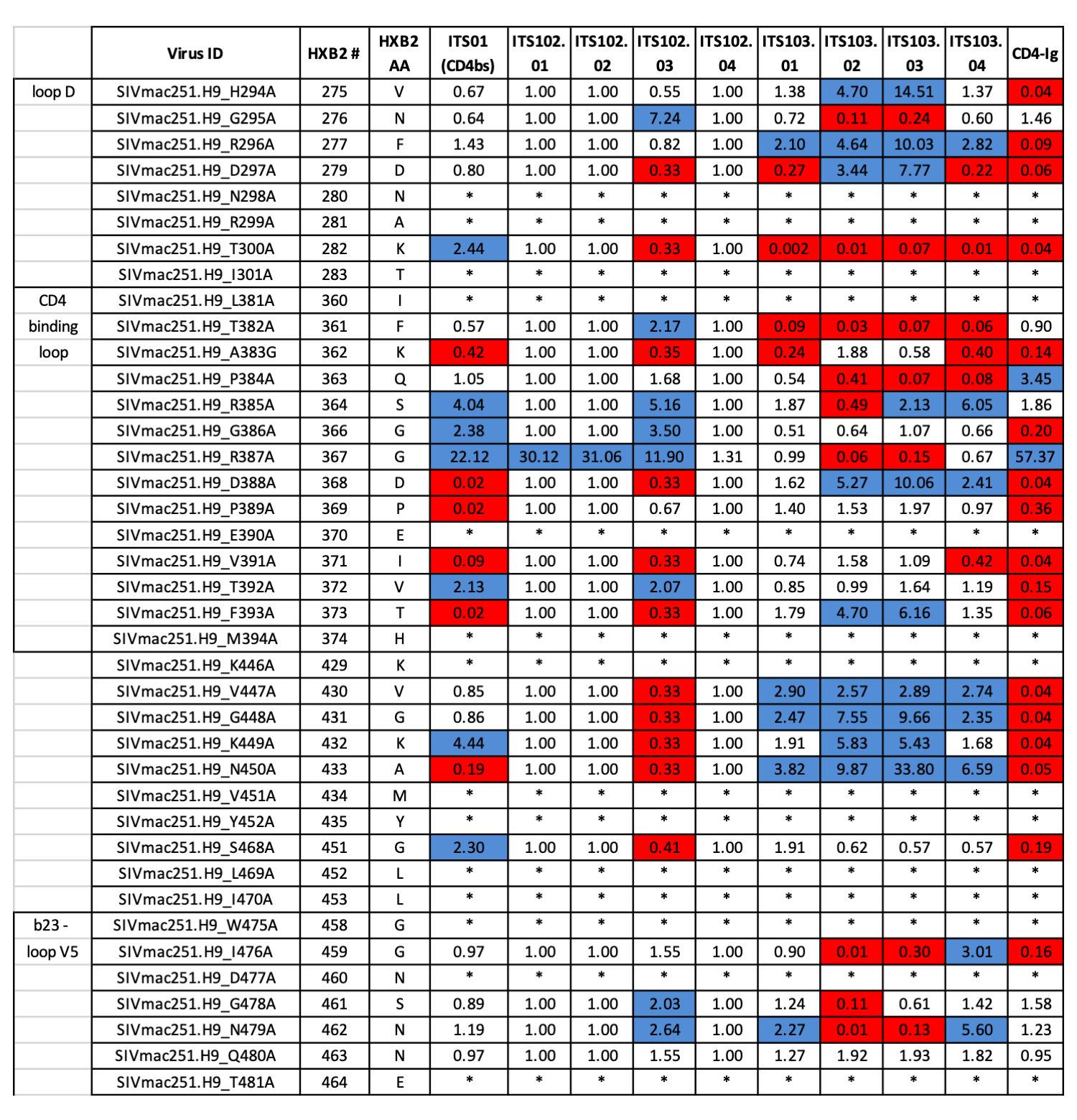

Supplement: S7 Fig — A panel of SIVmac251.H9.15 alanine scanning Env pseudo-typed virus mutants were used to map the neutralizing activity of SIV bnAbs. IC50 WT / IC50 mutant ratios < 0.5 and > 2 indicate increased sensitivity (red) or resistance (blue), respectively relative to wild-type SIVmac251.H9.15. Viruses marked with an asterisk (*) indicates viruses not tested due to low/absent infection. (TIF) [file ppat.1010574.s009.tif]

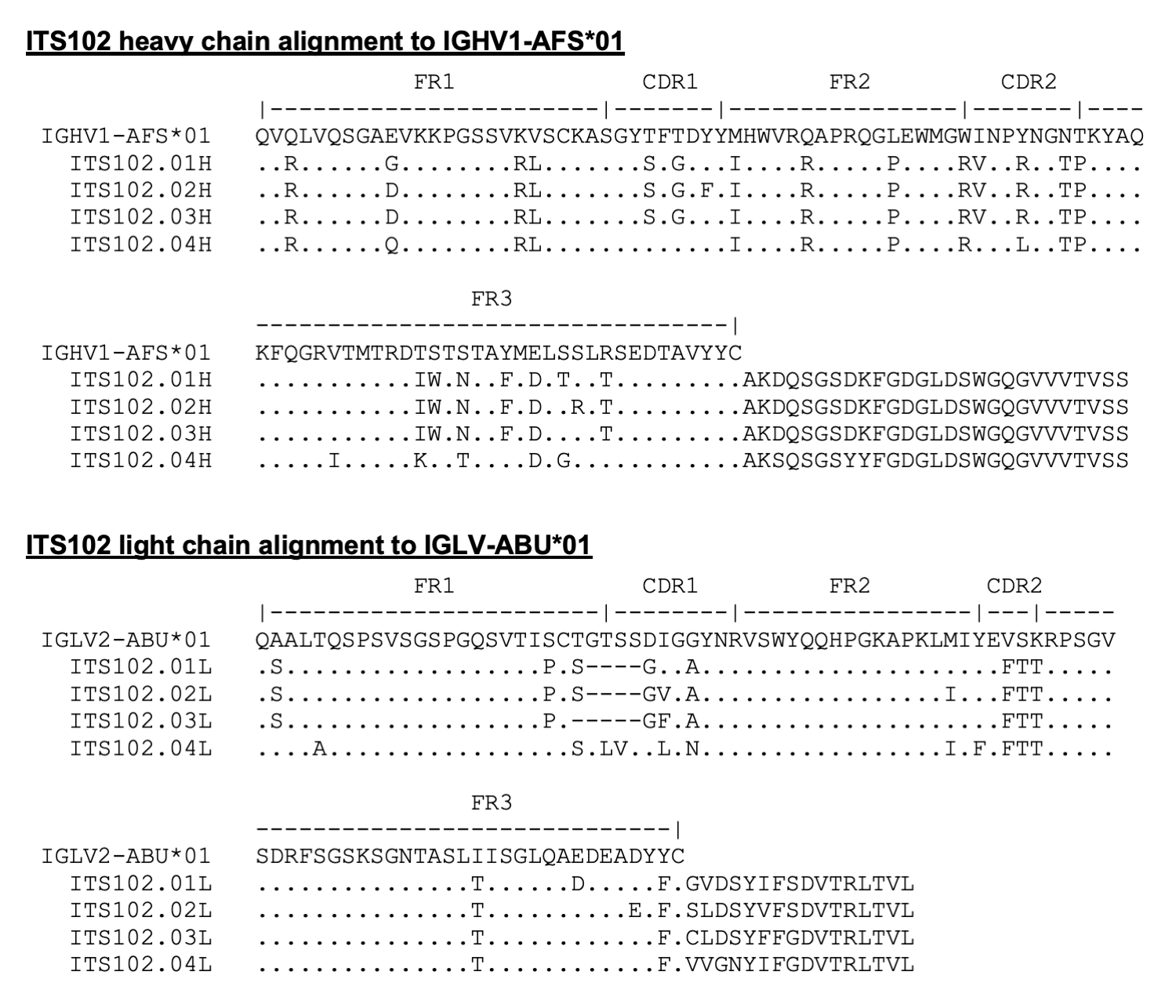

Supplement: S8 Fig — Alignment of the deduced amino acid sequences of the variable regions of ITS102 bnAbs to predicted germline IGHV and IGLV genes. Framework (FR) and CDRs are indicated above each sequence alignment. (TIF) [file ppat.1010574.s010.tif]

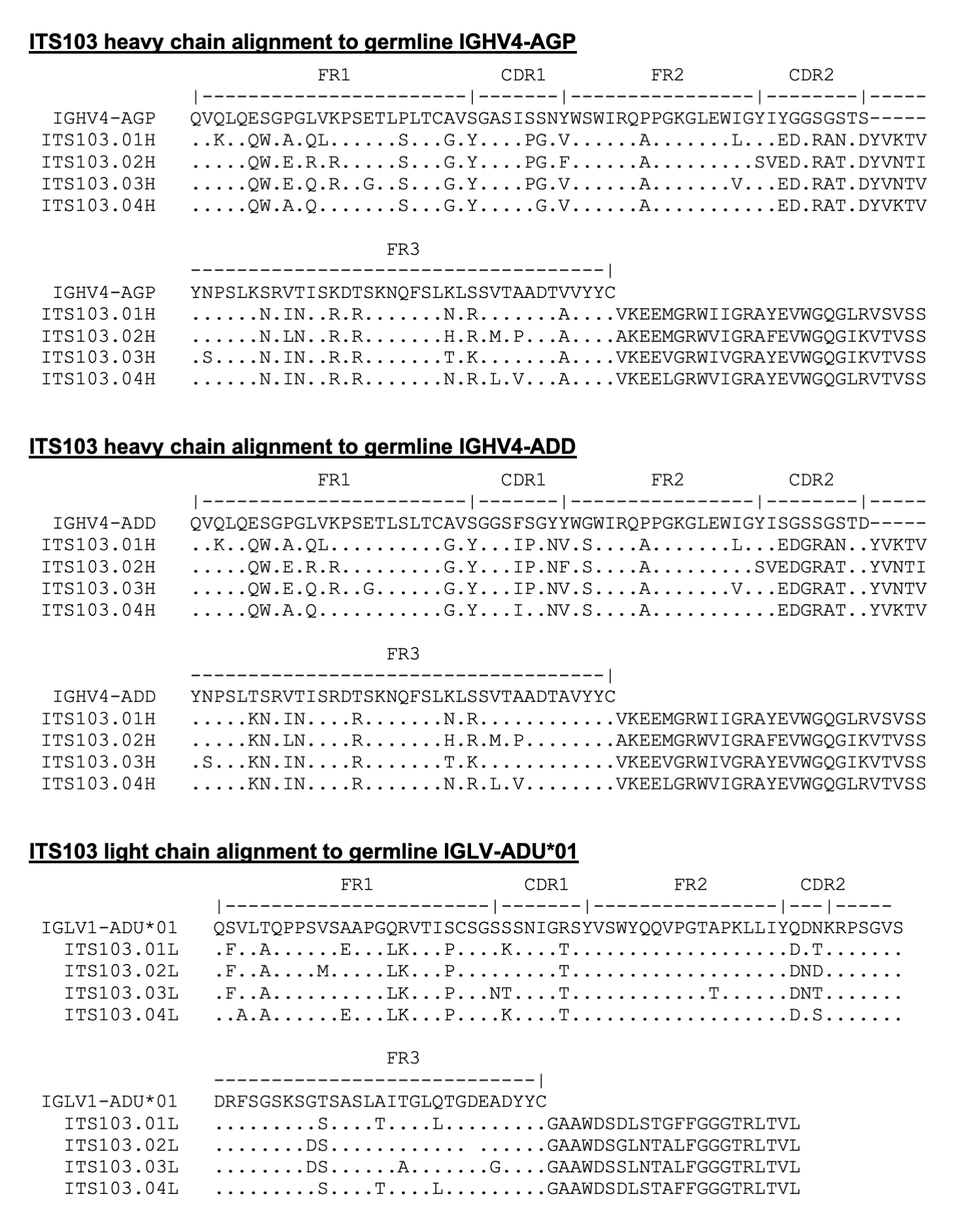

Supplement: S9 Fig — Alignment of the deduced amino acid sequences of the variable regions of ITS103 bnAbs to predicted germline IGHV and IGLV genes. Framework (FR) and CDRs are indicated above each sequence alignment. (TIF) [file ppat.1010574.s011.tif]

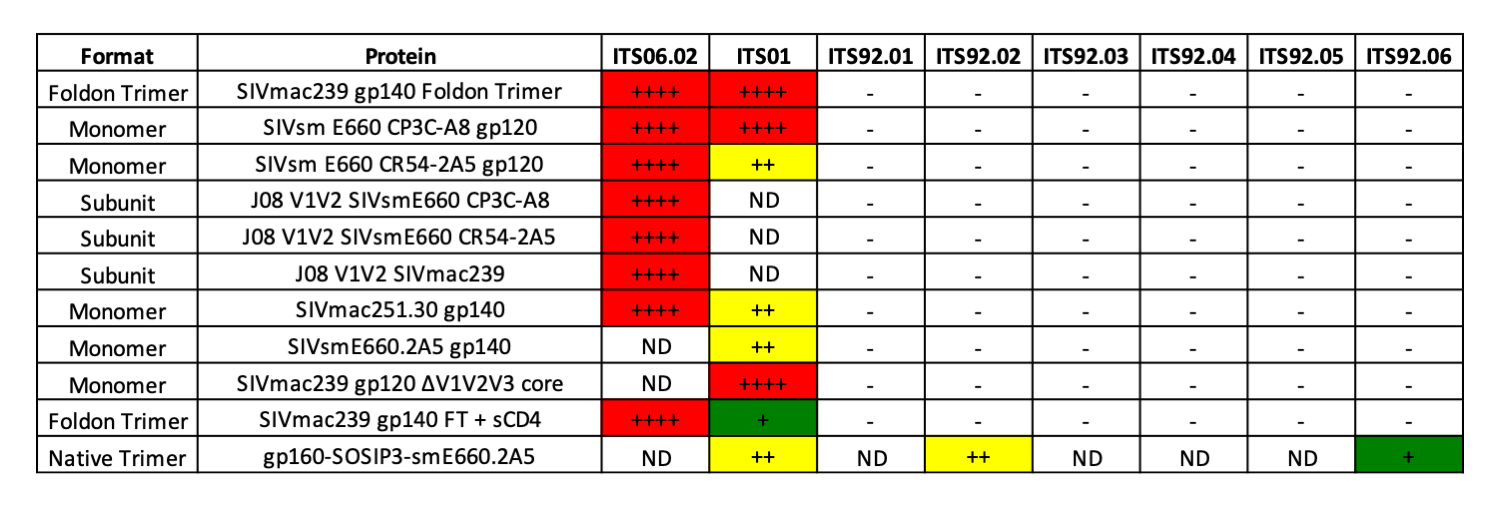

Supplement: S10 Fig — ITS92 family antibodies were tested for binding to various SIV proteins. Binding avidity is expressed as follows: ++++, OD450 ≥ 3.0 and EC50 ≤ 0.1 μg/ml; +++, OD450 ≥ 3.0 and EC50 > 0.1 μg/ml; ++, 1.0 ≤ OD450 < 3.0; +, 0.2 ≤ OD450 < 1.0. (TIF) [file ppat.1010574.s012.tif]

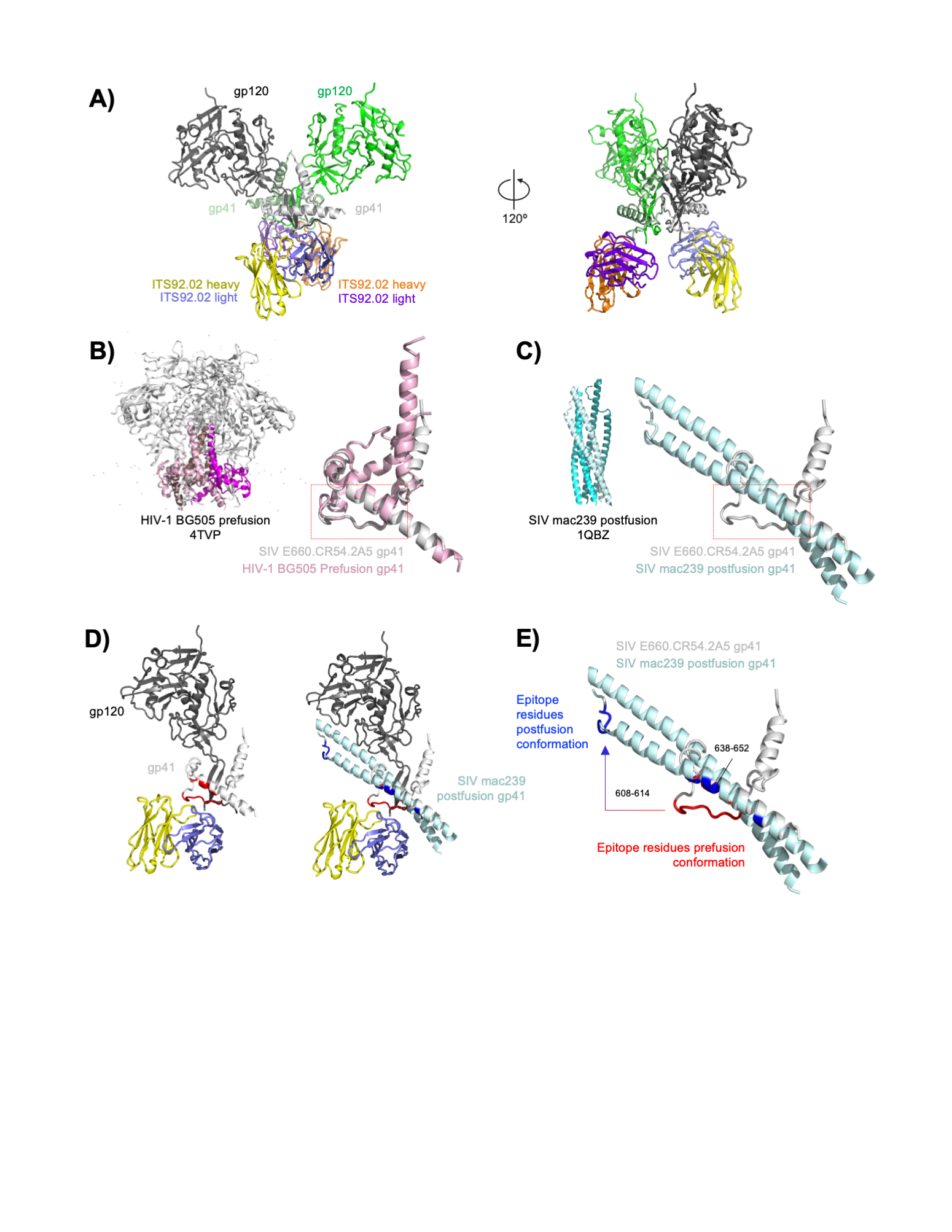

Supplement: S11 Fig — A) ITS92.02 bound to a SIV E660.CR54.2A5 SOS-2P dimer is shown in two orientations in cartoon format. B) The HIV-1 BG505 SOSIP trimer (PDB ID 4TVP) is shown next to an overlay of one HIV-1 prefusion gp41 chain (pink) with a gp41 chain from the ITS92.02-bound dimer (grey). A red box highlights the well-aligned ITS92.02 epitope region. C) Postfusion gp41 from SIV mac239 (PDB ID 1QBZ) is shown next to a single gp41 chain (light cyan) aligned with a chain from the ITS92.02-bound dimer (grey). D) One protomer of the SIV E660.CR54.2A5 dimer is shown next to the aligned SIV mac239 postfusion conformation. E) The gp41 chain of prefusion and postfusion gp41 conformations are shown with a 5 Å footprint of the ITS92.02 epitope is shown in red for prefusion and blue for postfusion. Epitope residues within the 638–652 helical range align well while residues within the 608–614 loop extend distantly away. (TIF) [file ppat.1010574.s013.tif]

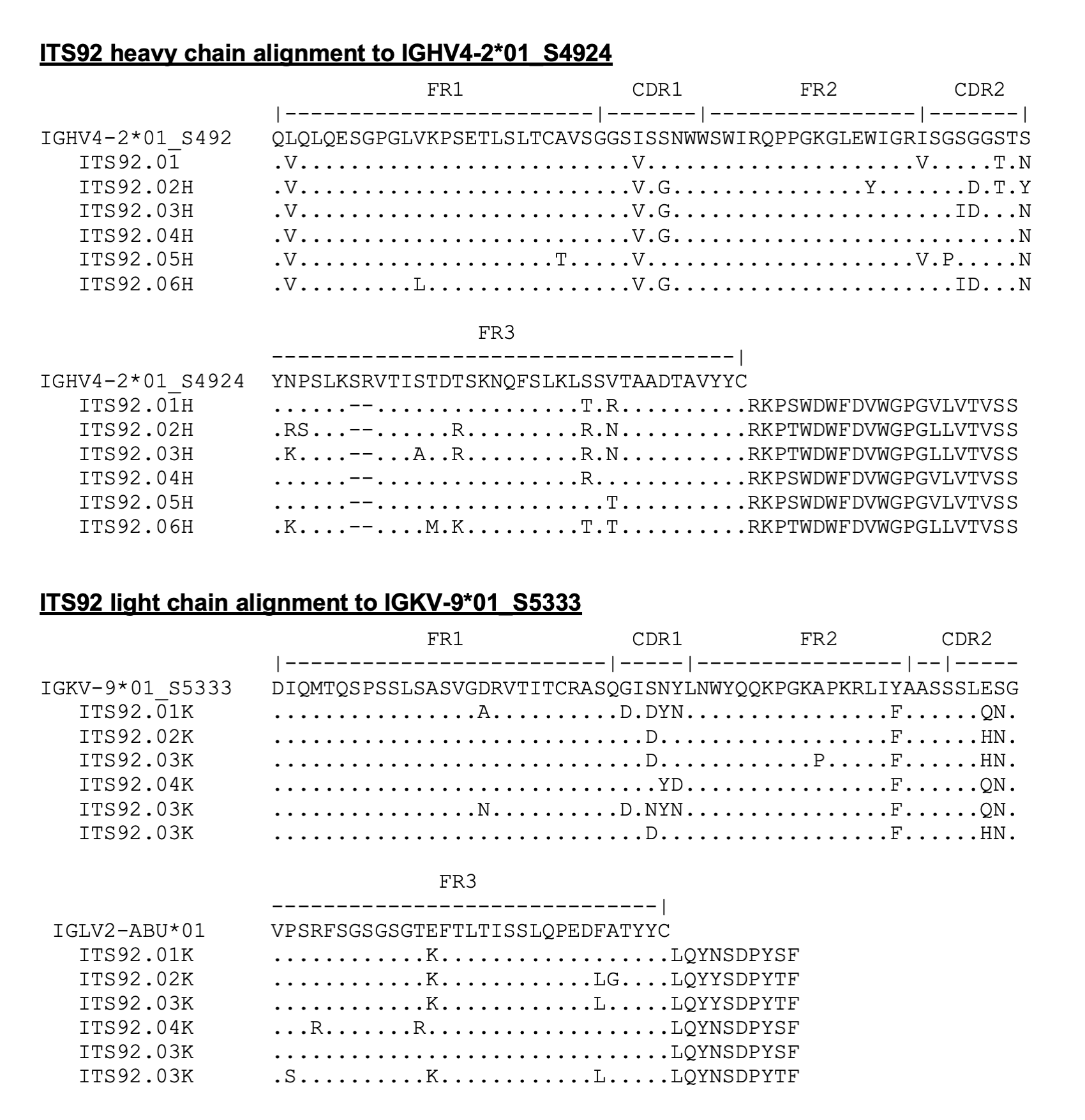

Supplement: S12 Fig — Alignments of the deduced amino acid sequences of the variable regions of ITS92 nAbs to predicted germline IGHV and IGKV genes. Framework (FR) and CDRs are indicated above each sequence alignment. (TIF) [file ppat.1010574.s014.tif]

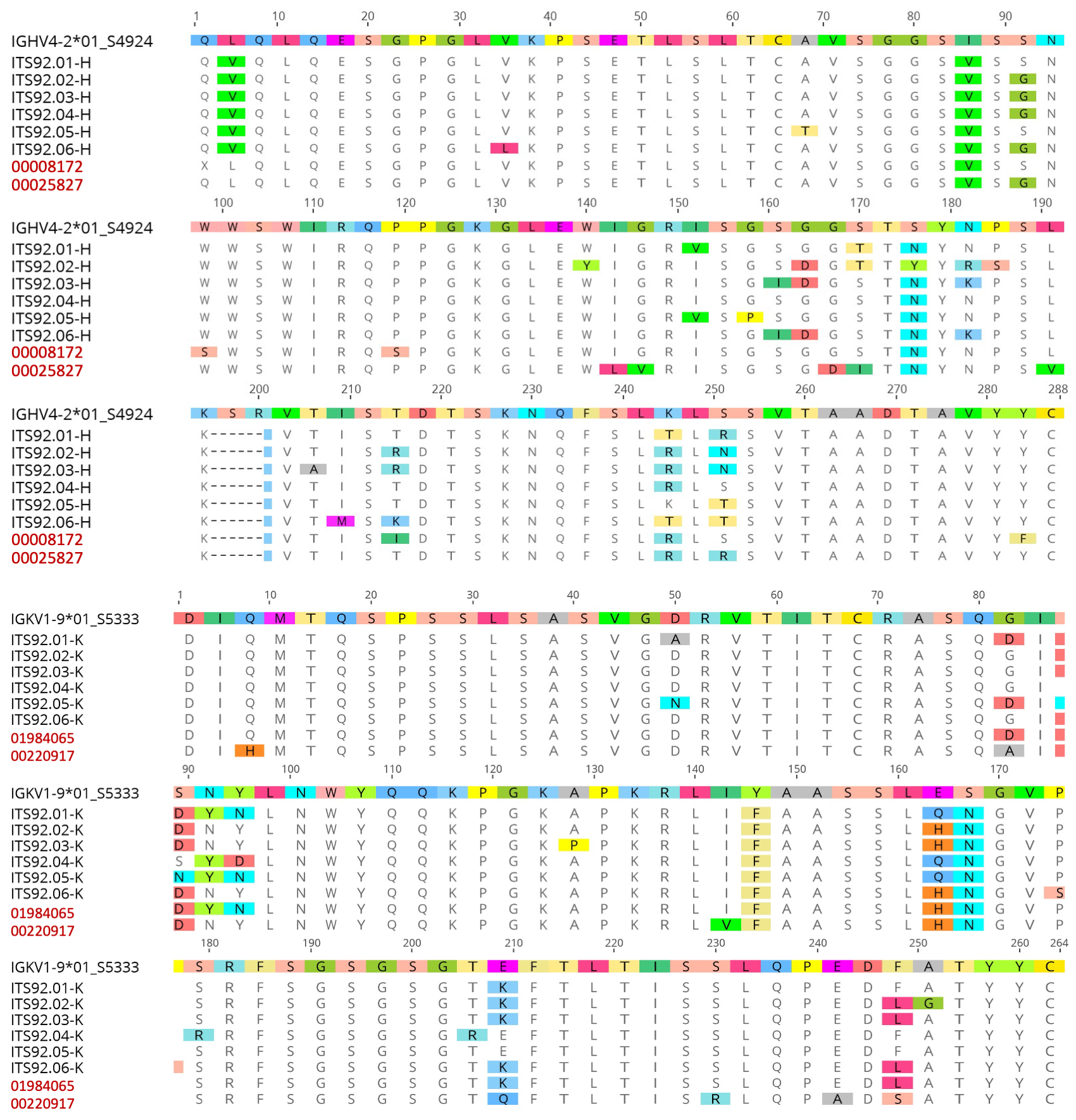

Supplement: S13 Fig — NGS data was probed for VH and VK regions with homology to ITS92 heavy and light chains. Alignments of VH (top) and VK (bottom) genes identified by NGS (red) to ITS92 and putative germline genes are displayed. (TIF) [file ppat.1010574.s015.tif]

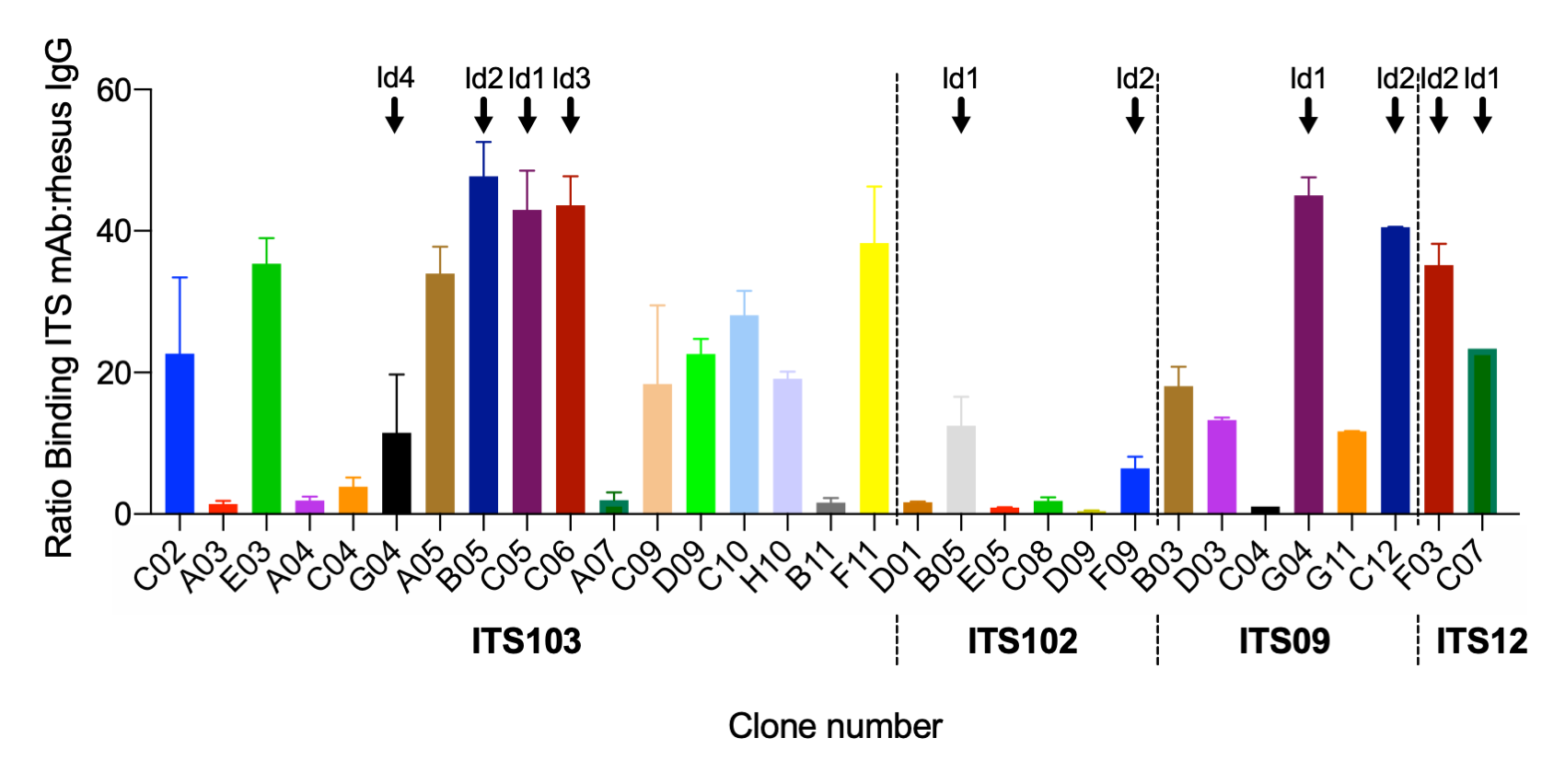

Supplement: S14 Fig — Binding of supernatant from transiently transfected 293F cells was assessed towards the specific ITS antibody (ITS103.01, ITS102.01, ITS09 or ITS12.01) and also to a pool of non-specific rhesus IgG by ELISA. ELISA plates were coated with 1 μg/mL ITS mAb, then cell culture supernatant from cells expressing anti-idiotype mAbs was used at a 1:1 dilution with buffer as the primary antibody. Shown here is the ratio of binding to this positive antibody and the negative rhesus IgG. X-axis labels indicate the anti-idiotype clone name, while labels above indicate names of the selected anti-Ids. (TIF) [file ppat.1010574.s016.tif]

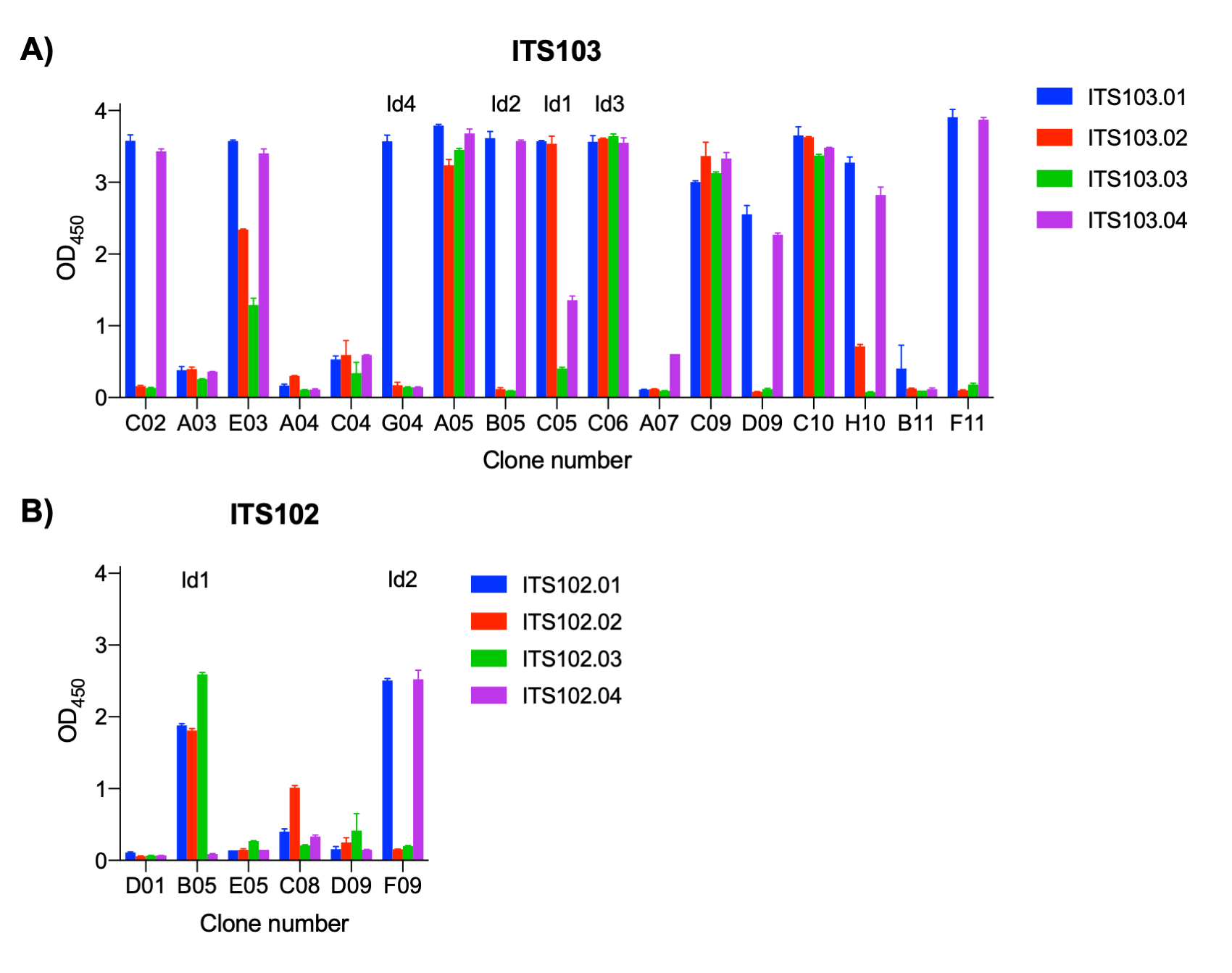

Supplement: S15 Fig — Binding of A) anti-ITS103 and B) anti-ITS102 mouse mAbs to different ITS103 (ITS103.01, ITS103.02, ITS103.03 and ITS103.04) and ITS102 (ITS102.01, ITS102.02, ITS102.03 and ITS102.04) clonal variants measured by ELISA. ELISA plates were coated with 1 μg/mL ITS clonal variants, then cell culture supernatant from cells expressing anti-idiotype mAbs was used at a 1:1 dilution with buffer as the primary antibody. (TIF) [file ppat.1010574.s017.tif]

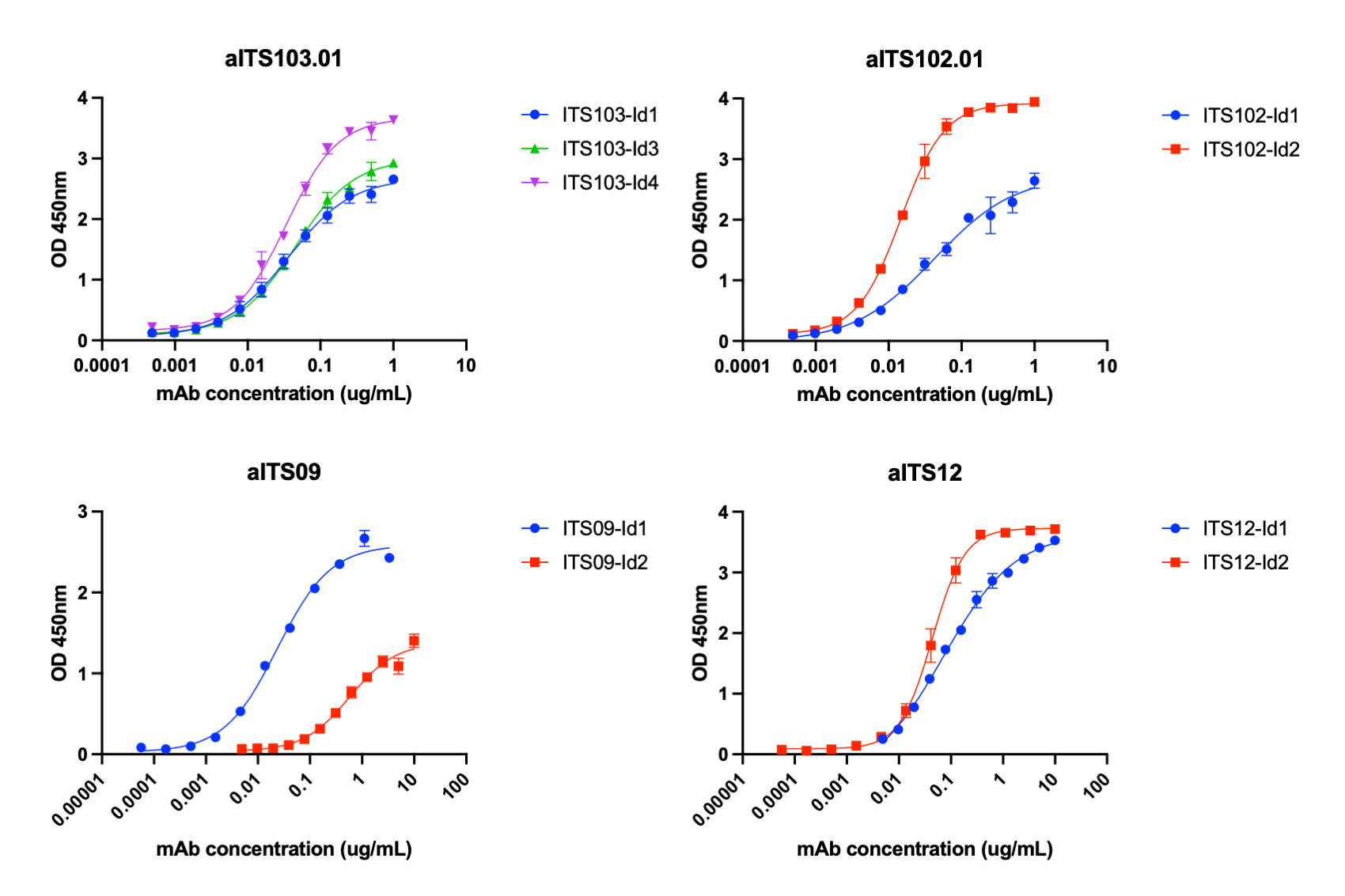

Supplement: S16 Fig — Binding of ITS mAbs to anti-idiotype mAbs was measured by ELISA. ELISA plates were coated with 1 μg/mL anti-idiotype mAb, and ITS mAbs were titrated at the concentration indicated. (TIF) [file ppat.1010574.s018.tif]
